# Targeting Ubiquitin‐Specific Protease 7 with Novel 5‐Amino‐Pyrazole Inhibitors: Design, Synthesis, and Biological Evaluation

**DOI:** 10.1002/cmdc.202500185

**Published:** 2025-06-24

**Authors:** Matteo Lusardi, Elva Morretta, Andrea Spallarossa, Maria Chiara Monti, Camillo Rosano, Erika Iervasi, Marco Ponassi, Matteo Mori, Fiorella Meneghetti, Chiara Brullo

**Affiliations:** ^1^ Department of Pharmacy Università degli Studi di Genova viale Benedetto XV, 3 16132 Genova Italy; ^2^ Molecular Modeling and Drug Discovery Laboratory Istituto Italiano di Tecnologia Via Morego, 30 16163 Genova Italy; ^3^ Department of Pharmacy University of Naples “Federico II” Via Domenico Montesano, 49 80131 Napoli Italy; ^4^ Proteomics and Mass Spectrometry Laboratory IRCCS Ospedale Policlinico San Martino L.go. R. Benzi, 10 I 16132 Genova Italy; ^5^ Department of Pharmaceutical Sciences University of Milan Via L. Mangiagalli, 25 20133 Milano Italy

**Keywords:** antiproliferative agents, computational simulations, pyrazole compounds, single crystal X‐ray diffraction, ubiquitin‐specific protease 7 inhibitors

## Abstract

To further extend the structure**–**activity relationships (SARs) of the previously published ubiquitin‐specific protease 7 (USP‐7) inhibitor **STIRUR‐41**, a small library of 5‐aminopyrazoles **1a–d** and **2a–d** is designed and synthesized. The chemical identity of the desired structure is confirmed by nuclear magnetic resonance and single crystal X‐ray diffraction analyses. All novel derivatives are tested as potential USP‐7 inhibitors and compounds **1a–d** block enzyme activity in a dose‐dependent manner and with lower IC_50_ values compared to the lead compound **STIRUR‐41**. Notably, **1d**, bearing a *meta*‐trifluoromethylphenyl group linked to the carbamate moiety, proved to be the most active candidate. Conversely, compounds belonging to series **2**, which possess greater steric hindrance, exhibit no activity. The most effective compounds of series **1** are noncytotoxic across a panel of tumor and normal cell lines at 10 μM concentration. For the most active compound **1d,** a parallel artificial membrane permeability assay is also performed, as well as docking and molecular dynamics simulations.

## Graphical abstract

Ubiquitin‐specific protease 7 (USP‐7) is a novel target for anticancer therapy. To further extend the structure–activity relationships of the previously USP‐7 inhibitor **STIRUR‐41**, a library of 5‐aminopyrazoles is reported. **1d** shows a potency comparable to lead compound **STIRUR‐41** and promising in silico and in vitro pharmacokinetic properties. Computational studies identify the molecular basis of USP‐7/1d interaction.

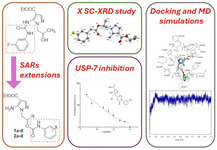

## 1 Introduction

The ubiquitin–proteasome system is the main protein regulatory system present in all eukaryotic cells, allowing the regulation, and consequently, elimination of all misfolded, aggregated, or unnecessary proteins, thus maintaining cellular homeostasis.^[^ ^1^ ^]^

**Figure 1:**
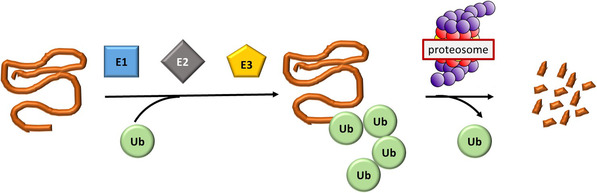
Schematic representation of the ubiquitin–proteasome system. Ub = ubiquitin. E1 = E1 ligase. E2 = E2 ligase. E3 = E3 ligase.

Protein ubiquitination is a post‐translational dynamics and reversible process that can be reversed by deubiquitinating enzymes (**Figure** **1**), called deubiquitinases (DUBs). Out of the seven DBU families, the ubiquitin‐specific protease 7 (USP‐7) family (also known as herpesvirus‐associated ubiquitin‐specific protease) is the largest.^[^ ^2^ ^]^ In the human genome, the USP‐7 family consists of 58 members that regulate many cellular proteins, including those involved in viral replication, immune response, epigenetic control, apoptosis, DNA damage response, and DNA replication and transcription. The role of USP‐7 in regulating these cellular pathways suggested this enzyme as a novel promising therapeutic target for oncology therapy.^[^ ^3^, ^4^ ^]^ Notably, elevated USP‐7 activity has been correlated with the onset, and subsequent progression of various human tumors through the interaction with oncogenic proteins, such as MDM2, MDMX,^[^ ^5^ ^]^ *β*‐catenin,^[^ ^6^ ^]^ NF‐κB,^[^ ^7^ ^]^ FOXP3,^[^ ^8^ ^]^ tumor suppressors p53, and PTEN.^[^ ^9^ ^]^ Furthermore, USP‐7 activity is also involved in cancer angiogenesis, facilitating the formation of new blood vessels within the tumor microenvironment.^[^ ^10^ ^]^ However, its role in tumor pathologies is dual, acting both as a promoter and as a tumor suppressor, depending on the cancer type.^[^ ^11^, ^12^ ^]^ In detail, USP‐7 overexpression has been observed in numerous malignant tumors, where it serves as a predictive marker of cancer progression and poor prognosis. In contrast, USP‐7 low expression has been identified in some neuroendocrine lung tumors^[^ ^13^ ^]^ and in nonsmall cell lung adenocarcinoma,^[^ ^14^ ^]^ suggesting that the role of USP‐7 can vary across different cancer types and may differently influence tumor behavior.

For all these reasons, in the last years, USP‐7 has emerged as a novel and exciting therapeutic target for cancer treatment, and several covalent and noncovalent small‐molecule inhibitors have been developed with promising efficacy in preclinical studies.^[^ ^15^, ^16^, ^17^, ^18^, ^19^, ^20^, ^21^, ^22^, ^23^ ^]^ Particularly, monocycles (e.g., thiazoles and pyridines) or bicyclic (e.g., quinazolines, pyrazolo‐pyrimidines, thieno‐pyrimidines, and imidazo‐triazines) and tricyclic derivatives (e.g., triterpenoid derivatives) have been recently published and patented.^[^ ^16^ ^]^

**Figure 2:**
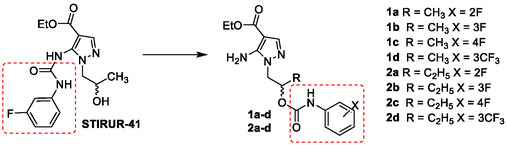
Molecular structure of the lead compound **STIRUR‐41** and the new 5‐amino carbamoyl derivatives **1a–d** and **2a–d**.

In this context, our previous investigations led to the identification of a large library of pyrazole derivatives able to blocks IL‐8‐induced chemotaxis of human neutrophils.^[^ ^24^ ^]^ In detail, the most active compound **STIRUR‐41** (**Figure** **2**) was submitted for additional investigations, showing a significant inhibition of migratory ability of neuroblastoma HTLA‐230 cells.^[^ ^25^ ^]^ Subsequent, drug affinity‐responsive target stability studies coupled to limited proteolysis‐coupled mass spectrometry, along with in vitro and in‐cell assays, recently demonstrated the ability of **STIRUR‐41** to inhibit both the enzymatic activity of USP‐7 and its expression levels in neuroblastoma‐related cells.^[^ ^26^ ^]^

To further extend the structure–activity relationship (SAR) of **STIRUR‐41,** the first‐in‐class pyrazole‐based USP‐7 inhibitor, 5‐aminopyrazoles (5APs) **1** and **2** (Figure 2) were designed and synthesized. Specifically, the phenylaminocarbonyl portion was formally shifted from the 5‐nitrogen atom of **STIRUR‐41** to the oxygen atom of the side chain, leading to the carbamate analogs **1** and **2**. The structure of the novel compounds was fine‐tuned by inserting fluorinated substituents (namely, F and CF_3_, selected on the basis of previously defined SARs) at different positions of the carbamate‐bound phenyl ring and by modifying the length of the side chain (i.e., 2‐hydroxypropyl and 2‐hydroxybutyl moieties).

We confirmed the formation of the desired structures by nuclear magnetic resonance (NMR) and X‐ray crystallographic analysis. Then, all newly synthesized compounds were evaluated for their in vitro USP‐7 inhibition. The most active compounds were also evaluated for their cytotoxicity (MTT assays at 10 μM concentration) in several cancer cell lines as well as in healthy human embryonic fibroblasts (GM‐6114) to verify that they did not affect cell viability, as the previous lead **STIRUR‐41**. Moreover, pharmacokinetics and toxicity properties as well as their drug‐likeness [SwissADME and ProTox web server] were calculated. Finally, parallel artificial membrane permeability assay (PAMPA) analysis was performed as well as docking and molecular dynamics simulations.

## 2 Results and Discussion

### 2.1 Chemistry

**Scheme 1:**
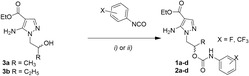
Synthesis of the new compounds **1a–d** and **2a–d**. i) Proper phenyl isocyanate, an. DMF, 100 °C, 12 h (yield: 23%–43%) and ii) excess of proper phenyl isocyanate, an. toluene, microwave, 100 °C, 300 W, 2 × 7 min (yield: 16%–27%).

Compounds **1** and **2** were obtained starting from the 5‐aminopyrazole intermediates **3a,b**, synthesized as previously reported (**Scheme** **1**).^[^ ^24^ ^]^ Briefly, the condensation of **3a** with the properly substituted isocyanate in anhydrous DMF at 100 °C for 12 h led to the isolation of compounds **1a–d** in moderate yields (23%–53%). Unfortunately, under these reaction conditions, 5‐aminopyrazole **3b** proved to be unreactive toward isocyanates, possibly due to the increase of the steric hindrance of the ethyl group adjacent to the alcohol functionality. Therefore, compounds **2a–d** were obtained through an alternative synthetic procedure involving the use of anhydrous toluene as a solvent, an excess of isocyanate reagent (3 equivalents), and two cycles of microwave heating (300 W, 7 min). With both methods, the final compounds were isolated through column chromatography (silica gel) using dichloromethane and ethyl ether as eluents.

Interestingly, pyrazoles **3** bear two nucleophilic centers (i.e., the NH_2_ and OH groups) potentially able to react with the isocyanate reagents. However, the different reaction conditions were found to affect the regioselectivity of the condensation. In detail, as previously reported, the use of an apolar solvent (toluene) under conventional heating conditions oriented the reaction toward the amino group of **3**, leading to the isolation of urea derivatives.^[^ ^24^, ^27^ ^]^ Conversely, heating the toluene mixture under microwave irradiation with an excess of heterocumulene or using polar aprotic solvents (e.g., DMF) led to the preferential reaction of the alcohol functionality allowing the formation of carbamate compounds.^[^ ^28^ ^]^

**Figure 3:**
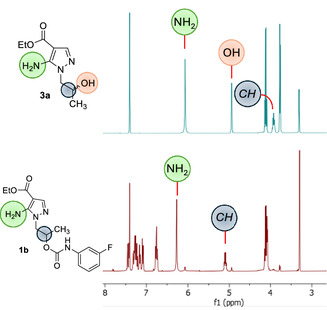
Comparison of the ^1^H NMR spectra of derivatives **3a** and **1b**. Relevant signals are highlighted.

The chemical identity of compounds **1** and **2** was determined by both NMR and X‐ray crystallography analyses. Specifically, the proton NMR signals at 6.00 ppm (present also in the spectra of precursors **3**) and the lack of the signal at 4.93 ppm (assigned to the OH in the spectra of **3**) indicated the presence of the free amino group at position 5 and confirmed the functionalization of the hydroxyl group in derivatives **1** and **2** (**Figure** **3**).

### 2.2 X SC‐XRD Study of 1b

**Figure 4:**
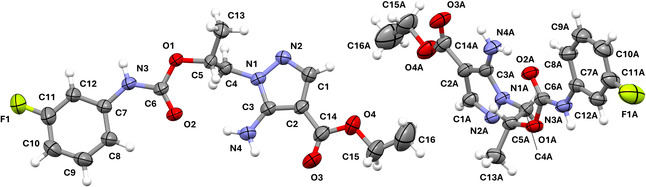
Thermal ellipsoid diagram of **1b**, with the arbitrary atom‐numbering scheme. Displacement ellipsoids are drawn at the 40% probability level.

The structure of **1b** was investigated by single crystal X‐ray diffraction (SC‐XRD) to confirm the success of the functionalization of the hydroxyl group and study the 3D features of this new class of 5‐APs. The compound crystallized in the orthorhombic system, with space group Pca2_1_. Its thermal ellipsoid diagram is shown in **Figure** **4**.

The asymmetric unit (ASU) contains two independent molecules, which overlay with a low root mean square deviation (RMSD) (0.1339 Å). The largest deviation (0.474 Å) occurs at the terminal methyl (C16) of the ethyl ester, reflecting minor structural flexibility. The analysis suggests a degree of pseudosymmetry, as the overall molecular arrangement nearly conforms to a higher‐symmetry space group, though the crystal ultimately refines in the noncentrosymmetric space group Pca2_1_. The compound contains a chiral center at C5/C5A. However, the Flack parameter converges to a meaningless value of −0.2(7) using Parsons’ method, likely due to a combination of factors, including the absence of anomalous scatterers and the pseudosymmetry, which increase the instability and uncertainty of the parameter. Therefore, despite this deviation from the ideal value, the molecule appears to be a racemate. The compound consists of two nearly parallel planar regions, corresponding to the substituted aminopyrazole and the phenyl carbamate moieties. These regions are connected by a methylene and a methine carbon atoms, forming dihedral angles of −164.4(3)° for N1—C4—C5—O1 and −164.9(3)° for N1A—C4A—C5A—O1A. The largest deviations from the mean planes calculated for the pyrazole and phenyl ring are observed in the ethyl ester, specifically at C16 (0.43 Å) and C15A (0.23 Å), and in the carbamate's alkoxy oxygen, at O1 (0.23 Å) and O1A (0.20 Å), respectively. The two planes are inclined at 8.4° and 10.7° with respect to each other in the number‐labeled and letter‐labeled molecules of the ASU, respectively.

**Figure 5:**
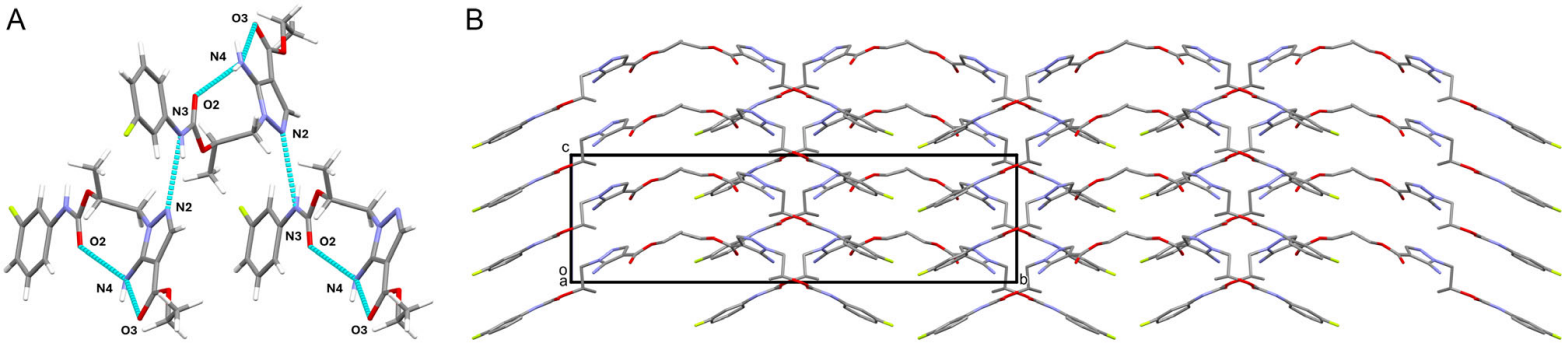
A) Stick model of **1b** showing the main H‐bonds as cyan dotted lines. B) Stick model showing the crystal packing of **1b**, viewed along the *a* axis. For the sake of clarity, hydrogen atoms are omitted.

The structure is characterized by strong intramolecular H‐bonds, connecting the amino group (N4, N4A) to the two carbonyl oxygens (O2 and O3, O2A and O3A) of the carbamate and ester moieties (**Figure** **5**A). These interactions play a key role in defining the molecular conformation, leading to atypical angles and torsions, as confirmed by the CSD Mogul analysis. Specifically, the previously mentioned dihedral angles deviate significantly from the typical range (45°–90°). Likewise, the C4—N1—C3 (126.5(3)°) and C4A—N1A—C3A (126.4(3)°) angles are notably smaller than the average value observed in similar structures (130.534°). The intermolecular network is also largely governed by H‐bonds. The main one connects the carbamate NH (N3/N3A) to one of the nitrogen atoms (N2/N2A) of the pyrazole ring of an adjacent molecule. Additional weaker interactions are formed between the aromatic CH groups and the N, O, and F atoms of nearby molecules. A complete account of H‐bonds is provided in **Table** **1**. π‐π Stacking contributes marginally to the crystal packing, with an inclined interaction between the phenyl groups, characterized by a centroid–centroid distance of 4.46 Å. Overall, the spatial organization of the crystal follows a double offset zig‐zag pattern (Figure 5B).

**Table 1:** H‐bond geometry (D: donor, a: acceptor) for 1b.

	D‐H/Å	H···A/Å	D···A/Å	D‐H···A/°	Equivalent positions
N4—H4W···O2^I^	0.860(3)	2.422(3)	3.210(4)	152.7(2)	^I^x, y, z; ^II^1‐x, 2‐y, z‐1/2; ^III^2‐x, 1‐y, z‐1/2; ^IV^x‐1/2, 1‐y, z; ^V^1‐x, 1‐y, z‐1/2; ^VI^x, y + 1, z; ^VII^2‐x, 2‐y, z‐1/2; ^VIII^x, y‐1, z
N4—H4X···O3^I^	0.860(3)	2.428(4)	2.957(5)	120.3(2)	
N4A—H4Y···O2A^I^	0.860(3)	2.451(3)	3.250(4)	154.8(3)	
N4A—H4Z···O3A^I^	0.860(3)	2.440(4)	2.969(5)	120.4(3)	
C8—H8···O2^I^	0.930(4)	2.310(3)	2.911(5)	121.9(3)	
C8A—H8A···O2A^I^	0.930(5)	2.335(3)	2.931(6)	121.6(3)	
N3A—H3A···N2A^II^	0.860(3)	2.181(3)	3.023(4)	166.2(2)	
C12A—H12A···N2A^II^	0.930(4)	2.844(3)	3.582(5)	137.2(3)	
C12—H12···N2^III^	0.930(4)	2.834(3)	3.567(5)	136.5(3)	
N3—H3···N2^III^	0.860(3)	2.248(3)	3.070(4)	160.0(2)	
N4—H4X···O1^IV^	0.860(3)	2.939(3)	3.615(4)	136.9(2)	
C9—H9···O3^V^	0.930(5)	2.517(3)	3.431(6)	167.7(3)	
C10A—H10A···F1^VI^	0.930(5)	2.655(4)	3.368(6)	134.1(3)	
C9A—H9A···O3A^VII^	0.930(5)	2.466(3)	3.385(6)	169.8(3)	
C10—H10···F1A^VIII^	0.930(6)	2.586(3)	3.499(6)	167.0(3)	

**Figure 6:**
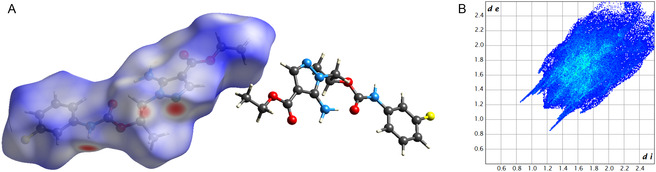
A) HS‐1 of **1b** mapped over *d*_*norm*_ with a fixed color scale in the range −0.4051 au (red)–1.6347 au (blue). Red, blue, and white indicate intermolecular contacts shorter, longer, and approximately equal to the sum of their van der Waals radii, respectively. B) 2D Fingerprint plot of **1b**. Each point represents a unique de/di pair, and its color indicates its contribution to the surface area: blue for low contribution, green for moderate, and red for high. Uncolored points have no contribution.

The Hirshfeld surface (HS) was computed for both molecules in the ASU, revealing similar features (HS‐1, V: 439.72 Å^3^, A: 399.52 Å^2^, *G*: 0.700, *Ω*: 0.440; HS‐A, V: 437.58 Å^3^, A: 401.30 Å^2^, *G*: 0.695, *Ω*: 0.458). The HS mapped over the *d*_*norm*_ property exhibits several red spots, both intense and faint, corresponding to the numerous traditional and nontraditional (CπH···N/O/F) H‐bonds established within the crystal structure (**Figure** **6**A). The 2D fingerprint plot has a compact and slightly elongated shape, with two spikes protruding toward the lower left part of the graph (short‐range region), at *d*_*e*_/*d*_*i*_ values compatible with NH···N bonds (Figure 6B). Shorter spikes, partially superposed to the previous ones, represent contact between aromatic hydrogens and heteroatoms (N/O/F). The plot also shows the presence of some stacking interactions (C–C contacts), although their contribution is marginal with respect to that of H‐bonds. The enrichment ratios confirm that N···H/H···N, O···H/H···O, F···H/H···F, and C···C contacts are favored, further corroborating the analysis of the intermolecular network (Table S1, Supporting Information).

### 2.3 Evaluation of Compound Effect on USP‐7 Enzymatic Activity

The inhibition efficiency of compounds **1a–d** and **2a–d** on the USP‐7 enzyme was assessed using a fluorescence‐based enzymatic assay, employing Ubiquitin–AMC as a substrate. In this assay, USP‐7 hydrolyzes Ubiquitin–AMC, leading to an increase in fluorescence signal. To evaluate the inhibitory potential, recombinant USP‐7 was preincubated with varying concentrations of the test compounds before adding the substrate, and fluorescence intensity was subsequently measured. The results revealed that compounds **1a–d** caused a reduction in fluorescence intensity, conceivably related to USP‐7 inhibition, in a dose‐dependent manner and with lower IC_50_ values compared to the lead compound **STIRUR‐41** (**Table** **2** and Figure S1, Supporting Information). Compound **1d**, characterized by a *meta*‐trifluoromethylphenyl group linked to the carbamate moiety, was the most active, while **1a**, which carries an *ortho*‐fluorine atom, proved to be the least potent. Additionally, the elongation of the side chain in compounds **2a–d** completely abolished the activity.

**Table 2:** IC_50_ values of compounds **1a‐d** and **STIRUR‐41** (used as reference compound) against recombinant USP‐7. The reported values are the average of three separate experiments.

Cpd	IC_50_ [μM] ± SD	*R* ^2^
**1a**	2.19 ± 0.49	0.873
**1b**	1.48 ± 0.31	0.889
**1c**	1.80 ± 0.39	0.879
**1d**	1.31 ± 0.21	0.932
**STIRUR‐41**	2.77 ± 0.56	0.947

### 2.4 Cytotoxicity

To evaluate the cytotoxicity of the novel USP‐7 inhibitors, compounds **1a–d** were preliminary screened at a fixed concentration of 10 μM against eight different cancer cell lines (namely, MCF7 breast adenocarcinoma, SKOV‐3 ovarian adenocarcinoma, Hep‐G2 hepatocellular carcinoma, SK‐Mel28 cutaneous melanoma, MDA‐MB‐231 breast adenocarcinoma, SK‐BR3 breast adenocarcinoma, HeLa cervix epithelioid carcinoma, and A549 lung adenocarcinoma) as well as nonmutated human embryonic fibroblasts (GM‐6114). Cisplatinum (*Cis*‐Pt) was used as the reference compound. As reported in **Table** **3**, compounds from series **1** were noncytotoxic across all tested cell lines (survival percentage values greater than 68%), with the unique exception of compound **1b**, which showed moderate cytotoxicity only against A549 cells (survival percentage = 39%). Collectively, **STIRUR‐41** and novel compounds **1a–d** showed a very similar antiproliferative profile, and interestingly, **STIRUR‐41** evidenced a major toxicity on healthy GM‐6114 cell lines. Consequently, we can hypothesize a better pharmacological profile of most active derivative **1d** respect to our lead **STIRUR‐41**.

**Table 3:** Cell growth percent values for compounds **1a‐d** and Cis‐Pt (reference) on different cancer cell lines at 10 μM concentration. Data are mean values of three separate experiments. Variation among triplicate samples is less than 10%.

					Cell growth percent [%]	
**Tumor cell line**	**1a**	**1b**	**1c**	**1 d**	**STIRUR‐41**	**Cis** **‐Pt**
**Lung adenocarcinoma**						
A549	104.43	39.06	114.96	90.69	38.88	59.09
**Cervix epithelioid carcinoma**						
HeLa	105.06	95.63	108.28	106.40	99.83	29.33
**Hepatocellular carcinoma**						
Hep‐G2	97.50	95.70	100.11	100.73	111.36	38.07
**Breast cancer**						
MCF7	109.46	104.08	108.02	113.85	110.71	72.74
MDA‐MB‐231	106.29	73.53	103.39	119.94	64.32	86.07
SK‐BR3	98.76	135.91	101.55	95.04	97.17	70.59
**Cutaneous melanoma**						
SK‐Mel28	95.28	115.09	88.22	90.77	94.26	44.40
**Ovarian adenocarcinoma**						
SKOV‐3	102.26	98.78	99.94	99.40	100.50	26.83
**Human embryonic fibroblasts**						
GM‐6114	105.96	68.07	112.09	96.18	38.50	39.52

### 2.5 Pharmacokinetic Properties, Drug‐Likeness, and Toxicity Prediction

The pharmaceutical, pharmacokinetic, and drug‐likeness properties of the most active compound **1d** and the lead **STIRUR‐41** were calculated by SwissADME.^[^ ^29^ ^]^ In the simulation, the two derivatives showed similar drug‐likeness and pharmacokinetic properties (Table S3, Supporting Information). Specifically, the fraction Csp^3^ was 0.35 and 0.31, the Log P (XLOGP3) values were 3.18 and 1.68, the number of rotatable bonds was 10 and 9, the number of H‐bond acceptors was 8 and 6, the number of H‐bond donors was 2 and 3, and the topological polar surface area was 108.47 and 105.48 Å^2^, for **1d** and **STIRUR‐41**, respectively. Altogether these compounds were predicted to be water soluble, characterized by high gastrointestinal absorption, and unable to cross the blood brain barrier. Differently to **STIRUR‐41**, **1d** was not flagged as a substrate for Pgp. In addition, no violations of the Lipinski rules were detected.

Finally, the toxicity profiles of **1d** and **STIRUR‐41** were predicted using ProTox webserver.^[^ ^30^ ^]^ According to the simulation, the predicted LD_50_ values for **STIRUR‐41** and **1d** were 1000 and 1550 mg Kg^−1^, respectively, being classified in the toxicity class 4. Interestingly, **1d** may only induce neurotoxicity, potentially emerging as a less toxic analog of **STIRUR‐41**, which is characterized by predicted neurotoxicity, nephrotoxicity, and respiratory toxicity.

### 2.6 PAMPA Assay

The membrane permeability of the most active derivative **1d** was determined using the PAMPA at two different concentrations (100 and 250 μM, respectively). Propranolol and furosemide were included as reference compounds for high and low permeability, respectively. The compound proved to be highly permeable at the tested concentrations, showing Log P_e_ values comparable to those of propranolol (**Table** **4**).

**Table 4:** LogP_e_ values of **1d**. Data are mean values of three separate experiments.

Sample (concentration)	LogP_e_ ± SD
Propranolol (250 μM)	−4.61 ± 0.01
Furosemide (250 μM)	−5.29 ± 0.02
**1d** (100 μM)	−4.54 ± 0.02
**1d** (250 μM)	−4.81 ± 0.00

### 2.7 Docking Simulations

**Figure 7:**
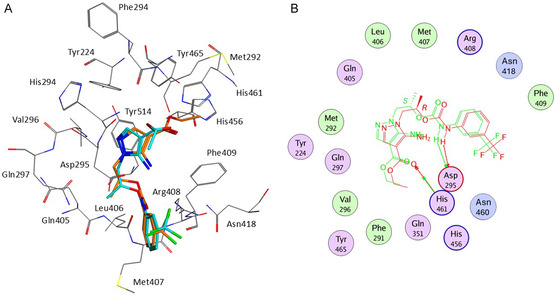
A) Docking poses of **1d(R)** (cyan) and **1d(S)** (orange) in USP‐7 binding site. B) Superposition of the log plot for the USP‐7/**1d(R)** and USP‐7/**1d(S)** complexes.

To further characterize the interaction between **1d** and USP‐7, docking simulations (Autodock4.2)^[^ ^31^ ^]^ were carried out on the USP‐7N‐terminal domain (PDB code: 5N9T).^[^ ^32^ ^]^ The docking protocol was validate through a redocking procedure on the cocrystallized ligand (RMSD value over heavy atoms = 0.474 Å; RMSD value over all atoms = 0.855 Å). Both enantiomers of the most active compound of the series were considered. In the USP‐7/**(** ***R*** **)‐1d** docking complex (calculated Ki = 2.85 μM; estimated free energy of binding = −7.56 kcal mol^−1^), the ligand would assume an extended conformation with the carbamate portion pointing toward the bulk solvent and the pyrazole moiety inserted into the binding site (**Figure** **7**).

The complex would be mainly stabilized by two hydrogen bonds, involving 1) the carbamate NH atom and Asp295 side chain and 2) the ester carbonyl and His461 side chain. Further, stabilization would be provided by Van Der Waals interactions between: 1) the pyrazole core and Tyr224 and Val296, 2) the carbon side chain and Asp295, Val296, Gln297, and Gln405, and 3) the 3‐CF_3_‐phenyl portion, and Phe409 and Asn418.

In the USP‐7/**(** ***S*** **)‐1d** docking complex, the ligand would adopt a similar bioactive conformation to that calculated for the (*R*)‐enantiomer. However, the different stereochemistry would affect the orientation of the pyrazole substructure, preventing the formation of the hydrogen bond between the ester functionality and His461. Despite the lack of this relevant interaction, the calculated Ki value of the USP‐7/**(** ***S*** **)‐1d** complex is slightly lower than that predicted for the USP‐7/**(** ***R*** **)‐1d** complex (calculated Ki = 2.51 μM; estimated free energy of binding = −7.64 kcal mol^−1^), thus, indicating that the (*S*) enantiomer should represent the eutomer.

### 2.8 Molecular Dynamics Simulations

**Figure 8:**
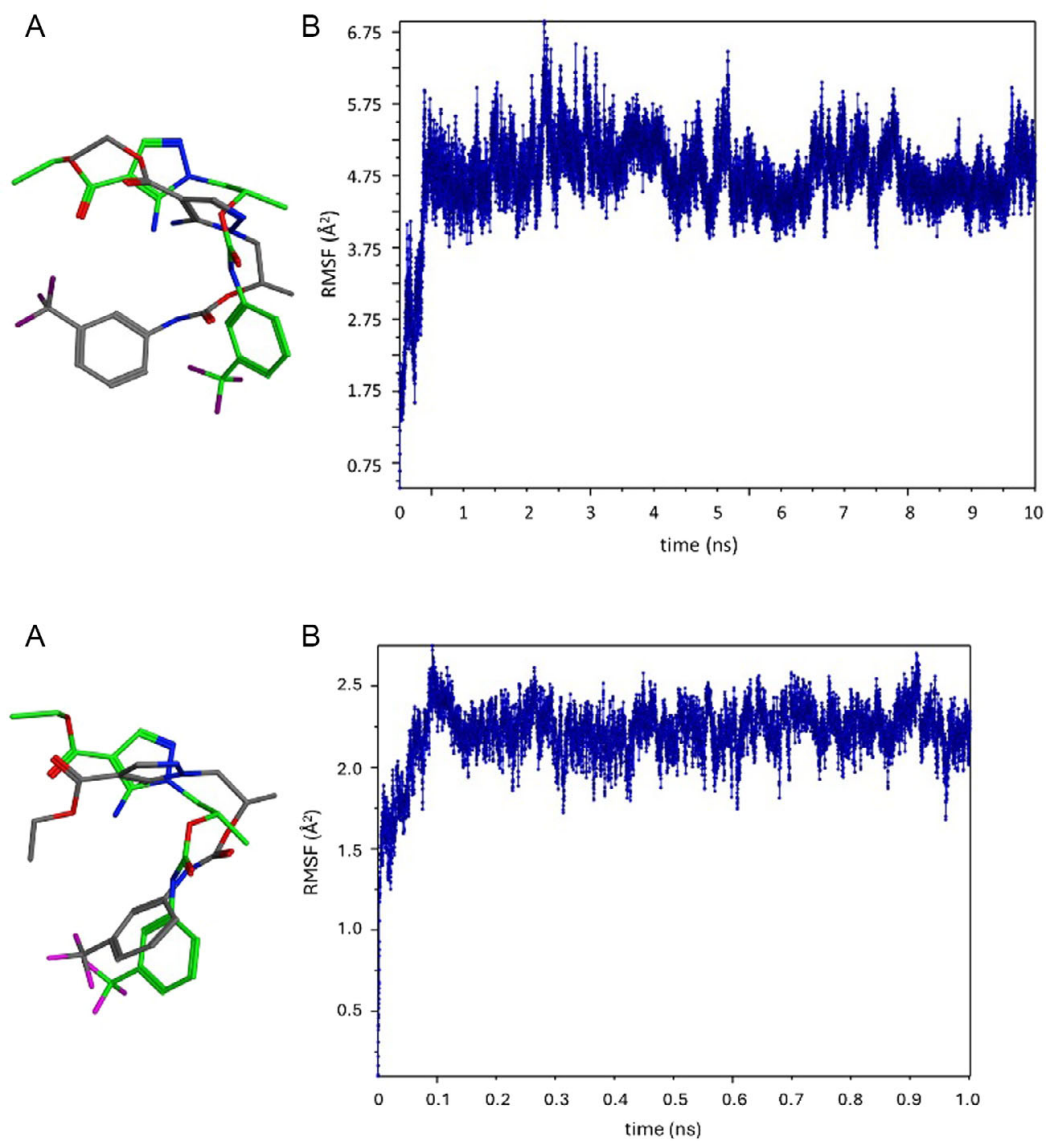
A) Superposition of the initial (green) and final (gray) bioactive conformation of **(** ***S*** **)‐1d**. B) Time‐dependent root‐mean‐square fluctuation analysis of ligand **(** ***S*** **)‐1d.**

To further asses the reliability of the docking calculations, a molecular dynamics simulation (NAMD software, explicit solvent, *T* = 310 K, time = 10 ns) was carried out on the USP‐7/**(** ***S*** **)‐1d** model, selected as the most promising docking complex. The molecular dynamics trajectory highlighted a stable binding between the ligand and the protein, with minimal fluctuations from the docking pose (**Figure** **8**). Furthermore, the analysis of the hydrogen bond network between **(** ***S*** **)‐1d** and USP‐7 residues indicated that the ligand could form one to three H‐bonds with the protein. In particular, the interaction between the carbamate NH or NH_2_ groups and Asp295 side chain would be the most likely H‐bond, being observed in the 16.54% of the MD conformations. Additional H‐bonds would involve the ligand and Arg408 main chain and carbamic carbonyl (frequency = 9.31%), Gln351 side chain and pyrazole nitrogen (frequency = 2.88%), and Tyr465 phenol group and ester carbonyl (frequency = 1.06%). Other amino acids H‐bonded to **(** ***S*** **)‐1d** would include Phe409 (0.33%), Leu406 (0.29%), Asn418 (0.26%), Tyr411 (0.19%), Val296 (0.06%), His461 (0.06%), and Gln297 (0.06%).

## 3 Conclusions

Collectively, the newly synthesized 5‐aminopyrazoles (5APs) **1a–d**, featuring a carbamate function on the N1 alkyl chain, demonstrated potent and dose‐dependent inhibition of USP‐7, achieving lower IC_50_ values than the lead compound **STIRUR‐41**. In contrast, compounds from series **2**, which possess greater steric hindrance, were completely inactive, highlighting the importance, as key element for an effective enzyme binding, of the structural flexibility.

The enzymatic data strongly support the role of specific structural modifications in enhancing USP‐7 inhibition. While a bulkier N1 alkyl chain negatively affects activity, the introduction of halogen atoms (especially fluorine) at different positions of the carbamate‐linked aromatic ring positively influences biological potency.

Collectively, the pyrazole derivatives reported here showed similar potency to other recently described monocyclic inhibitors (percentage of inhibition in the low micromolar range).^[^ ^16^ ^]^ Particularly, among the tested derivatives, compound **1d** emerged as the most promising, exhibiting strong USP‐7 inhibition and high membrane permeability (as confirmed by PAMPA). In addition, respect to the lead **STIRUR‐41,** **1d** showed lesser toxicity on healthy human embryonic fibroblasts GM‐6114 cell lines and a better predicted toxicity profile; consequently, we can hypothesize a better pharmacological profile of **1d** respect to our lead **STIRUR‐41**.

Although the precise role of USP‐7 in cancer is not yet fully elucidated, its key involvement in regulating major cancer hallmarks and oncogenic pathways makes it an attractive and high‐value target for cancer therapy.^[^ ^10^ ^]^ The identification of novel USP‐7 inhibitors endowed with an unreported chemical structure, low micromolar potency, excellent permeability, and absence of toxicity contributes to clarify the role of this enzyme in carcinogenesis and provide the basis for future studies to identify more effective USP‐7 pyrazole blockers.

## 4 Experimental Section

4.1

4.1.1

##### Chemistry

All the reagents were purchased by Alfa‐Aesar and Sigma‐Aldrich. DMF was reagent‐grade and was dried on molecular sieves (5 Å 1/16" inch pellets). Unless otherwise stated, all commercial reagents were used without further purification. Organic solutions were dried over anhydrous sodium sulphate. A thin layer chromatography system was utilized for routine monitoring of reaction progress and for confirming the purity of analytical samples, employing aluminum‐backed silica gel plates (Merck DC‐Alufolien Kieselgel 60 F254). Neat DCM or DCM/2% methanol mixture were used as the eluent, and detection of spots was achieved by UV light and/or by iodine vapors. Merck silica gel, 230–400 mesh, was used for chromatography. Flash chromatography was performed on an Isolera One instrument (Biotage, Uppsala, Sweden) using Silica gel columns. Melting points were determined on a BÜCHI M‐560 (Buchi instruments, Flawil, Switzerland) apparatus and were uncorrected. Microwave apparatus: CEM Discover (CEM Corporation, Matthews, NC, USA), a single‐mode microwave oven, with a max emitted power of 300 W, temperature‐controlled by an optical fiber, with magnetic stirring of the sample, and an air‐cooling system. ^1^H NMR and ^13^C NMR spectra were recorded on a JEOL JNMECZR (400 MHz, Tokyo, Japan). Chemical shifts were reported in δ (ppm) units relative to the internal reference tetramethylsilane, and the splitting patterns were described as follows: s (singlet), bs (broad singlet), d (doublet), t (triplet), q (quartet), and m (multiplet). The first order values reported for coupling constants *J* were given in Hz. Elemental analyzes were determined by an EA1110 Analyzer, Fison Instruments (Milan, Italy). Products were considered pure when the difference between calculated and found values is ± 0.4. Compounds **3a,b** were prepared as previously reported.^[^ ^24^ ^]^

##### General Synthetic Procedure for the Preparation of Compounds 1a‐b

To a dry DMF solution (5 mL) of **3a** (213 mg, 1 mmol), the proper phenyl isocyanate (1.1 mmol) was added dropwise under stirring at rt. The mixture was stirred at 100 °C for 12 h, then cooled to rt, and finally, added to water (30 mL). After alkalinization with NH_3_, the mixture was extracted with ethyl acetate (3 × 10 mL). The combined organic phases were washed with water (3 × 10 mL) and brine (3 × 10 mL), dried (MgSO_4_), and evaporated under reduced pressure. The crude product was purified by Flash Chromatography (Silicagel, eluent: DCM ‐ DCM/50% Et_2_O‐Et_2_O).

##### Ethyl 5‐Amino‐1‐(2‐(((2‐Fluorophenyl)Carbamoyl)Oxy)Propyl)‐1H‐Pyrazole‐4‐Carboxylate 1a

Yield: 33%. White solid. Mp 128–130 °C (DCM/Et_2_O). ^1^H NMR (400 MHz, DMSO‐*d*_6_) δ 1.19–1.26 (m, 6H, CHCH_3_+ CH_33_ ester), 4.06–4.18 (m, 4H, OCH_2_ + NCH_2_), 5.06–5.12 (m, 1H, CHO), 6.23 (bs, 2H, NH_2_, exchangeable), 7.09–7.22 (m, 3H, arom. H), 7.46 (s, 1H, H pyraz.), 7.51–7.57 (m, 1H, arom. H), and 9.21 (bs, 1H, NH, exchangeable). ^13^C NMR (101 MHz, DMSO‐*d*_6_) δ 163.45, 153.19, 150.39, 138.76, 126.02, 125.90, 125.32, 124.26, 115.67, 115.47, 93.79, 69.19, 58.66, 50.56, 17.56, and 14.52. Anal calcd. for C_16_H_19_FN_4_O_4_: C: 54.85; H: 5.47; N: 15.99. Found: C: 54.57; H: 5.27; N: 15.59.

##### Ethyl 5‐Amino‐1‐(2‐(((3‐Fluorophenyl)Carbamoyl)Oxy)Propyl)‐1H‐Pyrazole‐4‐Carboxylate 1b

Yield: 43%. White solid. Mp 144–145 °C (DCM/Et_2_O). ^1^H NMR (400 MHz, DMSO‐*d*_6_) δ 1.18–1.26 (m, 6H, CHCH_3_+ CH_3_ ester), 4.07–4.18 (m, 4H, OCH_2_ + NCH_2_), 5.08–5.17 (m, 1H, CHO), 6.31 (bs, 2H, NH_2_, exchangeable), 6.74–6.82 (m, 2H, arom. H), 7.26–7.34 (m, 2H, arom. H), 7.44 (s, 1H, H pyraz.), and 8.98 (bs, 1H, NH, exchangeable). ^13^C NMR (101 MHz, DMSO‐*d*_6_) δ 163.66, 152.89, 150.58, 141.15, 138.95, 130.61, 114.30, 109.11, 108.90, 105.05, 93.93, 69.03, 58.87, 50.70, 17.80, and 14.73. Anal calcd. for C_16_H_19_FN_4_O_4_: C: 54.85; H: 5.47; N: 15.99. Found: C: 54.67; H: 5.18; N: 15.89.

##### Ethyl 5‐Amino‐1‐(2‐(((4‐Fluorophenyl)Carbamoyl)Oxy)Propyl)‐1H‐Pyrazole‐4‐Carboxylate 1c

Yield: 53%. White solid. Mp 169–170 °C (DCM/Et_2_O). ^1^H NMR (400 MHz, DMSO‐*d*_6_) δ 1.20–1.25 (m, 6H, CHCH_3_+ CH_3_ ester), 4.08–4.18 (m, 4H, OCH_2_ + NCH_2_), 5.09–5.15 (m, 1H, CHO), 6.28 (bs, 2H, NH_2_, exchangeable), 7.01–7.12 (m, 2H, arom. H), 7.40–7.47 (m, 3H, arom. H + H pyraz.), and 9.60 (bs, 1H, NH, exchangeable). ^13^C NMR (101 MHz, DMSO‐*d*_6_) δ 163.96, 153.39, 150.87, 139.25, 135.95, 120.45, 115.89, 115.67, 94.24, 69.14, 59.18, 51.04, 18.16, and 15.04. Anal calcd for C_16_H_19_FN_4_O_4_: C: 54.85; H: 5.47; N: 15.99. Found: C: 54.50; H: 5.50; N: 16.26.

##### Ethyl 5‐Amino‐1‐(2‐(((3‐(Trifluoromethyl)Phenyl)Carbamoyl)Oxy)Propyl)‐1H‐Pyrazole‐4‐Carboxylate 1d

Yield: 43%. White solid. Mp 162–163 °C (DCM/Et_2_O). ^1^H NMR (400 MHz, DMSO‐*d*_6_) δ 1.20–1.26 (m, 6 H, CHCH_3_+ CH_3_ ester), 4.10–4.17 (m, 4H, OCH_2_ + NCH_2_), 5.11–5.18 (m, 1H, CHO), 6.30 (bs, 2H, NH_2_, exchangeable), 7.29–7.33 (m, 1H, arom. H), 7.44 (s, 1H, H pyraz.), 7.46–7.52 (m, 1H, arom. H), 7.62–7.66 (m, 1H, arom. H), 7.85–7.88 (m, 1H, arom. H), and 9.94 (bs, 1H, NH, exchangeable). ^13^C NMR (101 MHz, DMSO‐*d*_6_) δ 163.95, 153.30, 150.91, 140.51, 139.25, 130.47, 129.85, 122.39, 119.26, 114.72, 94.23, 69.48, 59.16, 51.00, 18.07, and 15.02. Anal calcd. for C_17_H_19_F_3_N_4_O_4_: C: 51.00; H: 4.78; N: 13.99. Found: C: 50.76; H: 4.74; N: 13.69.

##### General Synthetic Procedure for the Preparation Of Compounds 2a‐d

To a dry Toluene solution (5 mL) of **3b** (227 mg, 1 mmol), the proper phenyl isocyanate (1.5 mmol) was added dropwise under stirring at rt. The mixture was stirred under microwave irradiation (max potency 300 W) at 90 °C for 7 min. Then, another equivalent of phenyl isocyanate (1.5 mmol) was added, and the solution was heated again under microwave irradiation at 90 °C for 7 min. After cooling to rt, the solvent was evaporated under reduced pressure, and the crude product was purified by Flash Chromatography (Silicagel, eluent: DCM ‐ DCM/50% Et_2_O‐Et_2_O).

##### Ethyl 5‐Amino‐1‐(2‐(((2‐Fluorophenyl)Carbamoyl)Oxy)Butyl)‐1H‐Pyrazole‐4‐Carboxylate 2a

Yield: 16%. White solid. Mp 97–98 °C (DCM/Et_2_O). ^1^H NMR (400 MHz, DMSO‐*d*_6_) δ 0.87 (t, *J* = 7.4 Hz, 3 H, CH_3_), 1.19 (t, *J* = 7.1 Hz, 3H, CH_3_ ester), 1.48–1.72 (m, 2 H, CHCH_2_), 4.04–4.14 (m, 4H, OCH_2_ + NCH_2_), 4.91–4.97 (m, 1H, CHO), 6.17 (bs, 2H, NH_2_, exchangeable), 7.05–7.18 (m, 3H, arom. H), 7.41 (s, 1H, H pyraz.), 7.48–7.54 (m, 1H, arom. H), and 9.18 (bs, 1H, NH, exchangeable). ^13^C NMR (101 MHz, DMSO‐*d*_6_) δ 163.97, 154.00, 150.93, 139.25, 126.63, 126.52, 125.71, 124.81, 124.78, 116.18, 94.34, 73.93, 59.18, 49.72, 24.93, 15.04, and 9.73. Anal calcd. for C_17_H_21_FN_4_O_4_: C: 56.04; H: 5.81; N: 15.38. Found: C: 55.70; H: 5.70; N: 15.45.

##### Ethyl 5‐Amino‐1‐(2‐(((3‐Fluorophenyl)Carbamoyl)Oxy)Butyl)‐1H‐Pyrazole‐4‐Carboxylate 2b

Yield: 27%. White solid. Mp 132–133 °C (DCM/Et_2_O). ^1^H NMR (400 MHz, DMSO‐*d*_6_) δ 0.92 (t, *J* = 7.3 Hz, 3 H, CH_3_), 1.22 (t, *J* = 7.1 Hz, 3 H, CH_3_ ester), 1.48–1.72 (m, 2H, CHCH_2_), 4.04–4.18 (m, 4H, OCH_2_ + NCH_2_), 4.97–5.08 (m, 1H, CHO), 6.28 (bs, 2H, NH_2_, exchangeable), 6.71–6.82 (m, 1H, arom. H), 7.15–7.36 (m, 3H, arom. H), 7.44 (s, 1H, H pyraz.), and 9.83 (bs, 1H, NH, exchangeable). ^13^C NMR (101 MHz, DMSO‐*d*_6_) δ 163.47, 161.14, 152.96, 150.44, 141.13, 138.71, 130.24, 114.07, 108.62, 104.84, 93.81, 73.12, 58.64, 49.18, 24.42, 14.47, and 9.24. Anal calcd. for C_17_H_21_FN_4_O_4_: C: 56.04; H: 5.81; N: 15.38. Found: C: 56.24; H: 5.76; N: 15.55.

##### Ethyl 5‐Amino‐1‐(2‐(((4‐Fluorophenyl)Carbamoyl)Oxy)Butyl)‐1H‐Pyrazole‐4‐Carboxylate 2c

Yield: 21%. White solid. Mp 126–127 °C (DCM/Et_2_O). ^1^H NMR (400 MHz, DMSO‐*d*_6_) δ 0.91 (t, *J* = 7.3 Hz, 3H, CH_3_), 1.23 (t, *J* = 7.1 Hz, 3H, CH_3_ ester), 1.48–1.68 (m, 2H, CHCH_2_), 4.09–4.17 (m, 4H, OCH_2_ + NCH_2_), 4.96–5.03 (m, 1H, CHO), 6.27 (bs, 2H, NH_2_, exchangeable), 7.06–7.12 (m, 2H, arom. H), 7.39–7.45 (m, 3H, arom. H + H pyraz.), and 9.63 (bs, 1H, NH, exchangeable). ^13^C NMR (101 MHz, DMSO‐*d*_6_) δ 163.44, 153.14, 150.39, 138.68, 135.47, 119.89, 115.36, 115.14, 93.75, 72.87, 58.65, 49.17, 24.43, 14.51, and 9.26. Anal calcd. for C_17_H_21_FN_4_O_4_: C: 56.04; H: 5.81; N: 15.38. Found: C: 56.30; H: 5.59; N: 15.14.

##### Ethyl 5‐Amino‐1‐(2‐(((3‐(Trifluoromethyl)Phenyl)Carbamoyl)Oxy)Butyl)‐1H‐Pyrazole‐4‐Carboxylate 2d

Yield: 24%. White solid. Mp 148–149 °C (DCM/Et_2_O). ^1^H NMR (400 MHz, DMSO‐*d*_6_) δ 0.92 (t, *J* = 7.4 Hz, 3H, CH_3_), 1.22 (t, *J* = 7.1 Hz, 3H, CH_3_ ester), 1.50–1.71 (m, 2H, CHCH_2_), 4.10‐4.17 (m, 4H, OCH_2_ + NCH_2_), 5.00–5.07 (m, 1H, CHO), 6.28 (bs, 2H, NH_2_, exchangeable), 7.29–7.33 (m, 1H, arom. H), 7.43 (s, 1H, H pyraz.), 7.46–7.51 (m, 1H, arom. H), 7.62–7.66 (m, 1 H, arom. H), 7.85–7.89 (m, 1 H, arom. H), and 9.96 (bs, 1H, NH, exchangeable). ^13^C NMR (101 MHz, DMSO‐*d*_6_) δ 163.23, 152.85, 150.23, 139.84, 138.48, 129.71, 129.14, 121.64, 118.47, 113.98, 93.55, 73.04, 58.41, 48.95, 30.49, 24.17, 14.26, and 9.06. Anal calcd. for C_18_H_21_F_3_N_4_O_4_: C: 52.17; H: 5.11; N: 13.52. Found: C: 52.04; H: 4.98; N: 13.85.

##### X‐ray Crystallography

Diffraction‐quality crystals of **1b** were grown as colorless plates through slow evaporation of a chloroform/methanol solution of the compound. X‐ray intensity data were collected at 293(2) K using a Rigaku XtaLAB Synergy diffractometer (Rigaku, Tokyo, Japan), equipped with a microfocus PhotonJet Mo‐Kα source and a hybrid pixel array detector. The data were obtained through omega scans with a step size of 0.5° and an exposure time of 40 s per frame. A total of 21 414 Bragg reflections were recorded, revealing a metrically orthorhombic unit cell. Systematic absence analysis suggested the space group Pca2^1^. Intensity data were integrated and empirically corrected for Lorentz‐polarization and absorption effects using CrysAlisPro 1.171.42.58a (Rigaku Oxford Diffraction/Agilent Technologies UK Ltd.). The structure was solved by direct methods with SIR2019/3^[^ ^33^ ^]^ and refined through iterative full‐matrix least‐squares refinement on Fo2 and ΔF synthesis using SHELXL‐2019/3^[^ ^34^ ^]^ within the WinGX suite (WinGX v.2023.1).^[^ ^35^ ^]^ Hydrogen atoms were placed in calculated positions according to their expected geometries and were refined using a riding model with fixed isotropic thermal parameters (1.2 and 1.5 Ueq of the parent atom for aromatic/methylene/methine and methyl groups, respectively). Structural analysis was performed using PARST^[^ ^36^ ^]^ and Mercury 2024.3.0;^[^ ^37^ ^]^ graphical representations were generated with Mercury. HS generation was carried out with CrystalExplorer21.^[^ ^38^ ^]^ Crystal data and structure refinement details are shown in Table S2, Supporting Information.

Deposition Number 2 421 400 (for compound **1b**) https://www.ccdc.cam.ac.UK/services/structures?id=doi:10.1002/cmdc.202 500 185 contains the supplementary crystallographic data for this paper. These data are provided free of charge by the joint Cambridge Crystallographic Data Centre and Fachinformationszentrum Karlsruhe http://www.ccdc.cam.ac.UK/structures Access Structures service.

##### Ub‐AMC Assay

In vitro enzymatic inhibition was measured using the USP‐7 inhibitor screening assay kit (catalog #79,256, BPS Bioscience, San Diego, CA, USA) according to the manufacturer's instructions. Briefly, compound stock solutions were prepared in DMSO and diluted tenfold to obtain the working solutions. Then, these solutions and the vehicle control were added to the black 384‐wells plate, together with the USP‐7 enzyme, previously diluted as required by the manufacturer, and incubated at rt for 30 min. Then, the Ub‐AMC substrate was diluted 400‐fold and added to the plate, followed by incubation at rt for 30 min. The final DMSO amount in each well was 1%. The fluorescence signal (excitation = 350 nm, emission = 460 nm) was recorded using the Victor Nivo microplate reader (Revvity, Waltham, MA, USA. IC_50_ was calculated by subtracting Ub‐AMC background from each value. The results are expressed as means ± SEM of at least three independent experiments. For IC_50_ determination, the curves were obtained using GraphPad Prism 7 (San Diego, CA, USA).

##### Antiproliferative Activity

To perform the MTT assay, SK‐BR3 (breast adenocarcinoma, Biologic Bank and Cell Factory, IRCCS Policlinico San Martino, Genoa, Italy), MCF‐7 (breast adenocarcinoma, Biologic Bank and Cell Factory, IRCCS Policlinico San Martino, Genoa, Italy), SK‐Mel28 (skin melanoma, Biologic Bank and Cell Factory, IRCCS Policlinico San Martino, Genoa, Italy), Hep‐G2 (hepatocellular carcinoma, ATCC, Manassas, VA), SKOV‐3 (ovarian adenocarcinoma, ATCC, Manassas, VA), MDA‐MB‐231 (breast adenocarcinoma, Biologic Bank and Cell Factory, IRCCS Policlinico San Martino, Genoa, Italy), HeLa (cervical adenocarcinoma, Biologic Bank and Cell Factory, IRCCS Policlinico San Martino, Genoa, Italy), A549 (lung adenocarcinoma, ATCC, Manassas, VA), and GM‐6114 (normal human embryonic fibroblast, ATCC, Manassas, VA) cell lines were cultured in Dulbecco's modified eagle medium (DMEM added with 10% Fetal bovine serum, 2 mM Glutamine, and 1% pen‐strep) and incubated in a humidified environment at 37 °C with 5% CO_2_. Reagents were acquired from EuroClone (Milan, Italy). All chemical compounds were dissolved in DMSO to give a 10 mM stock solution. Then, after an intermediate dilution in growth medium, they were added to the cultured cells at a final working concentration of 10 μM and incubated for 48 h. At the end of the incubation, 30 μL of MTT (3‐(4,5‐dimethyl‐2‐thiazolyl)‐2,5‐diphenyl‐2H‐tetrazolium bromide) at a concentration of 2 mg mL^−1^ in phosphate buffered saline (PBS) were added into each well and further incubated for 4 h. Finally, the supernatants were removed and 100 μL well of DMSO were added to each well to dissolve the Formazan precipitates. After 20 min, plates were read at 570 nm. Results are expressed as the percentage of the control samples where cells have been treated with the same amount of DMSO but without any chemical compound. The assay was repeated three times, and a single compound was tested six times. Means and standard deviations were calculated.

##### PAMPA Assays

The assay was prepared as follows: 300 μL of PBS pH 7.4 (5% DMSO) was placed in the acceptor wells. A phosphatidylcholine (PC) solution was obtained by dissolving and vortexing PC at 1% w/v in dodecane and solubilized using an ultrasound bath until a clear solution was obtained. 5 mL of this solution was deposited on the membrane portion of the donor well. 150 μL of each molecule, at 100–250 μM final concentration (5% DMSO), was pipetted in the acceptor wells. The acceptor (Multiscreen transport Receiver Plate, 96‐well, polystyrene, Millipore) and donor plates (Multiscreen Filter Plates, IP, 0.45 mm, clear, hydrophobic PVDF, Millipore) were subsequently assembled and incubated for 5 h under gentle orbital shaking. The experiment was conducted in duplicate. Lucifer yellow was used as a negative control to establish the integrity of the membrane, quantifying its concentration in both acceptor and donor solutions using fluorescence. Propranolol and Furosemide were used as positive and negative controls. Acceptor, donor, and equilibrium solutions, along with the original 100–250 μM stock solutions, were analyzed by UV detector at 250 nm for compound **1d**, 280 nm for propranolol, and 275 nm for furosemide (Victor Nivo, Revvity, USA). The results are expressed as means ± SEM of at least three independent experiments.

##### Computational Studies

The calculations were run on a Linux PC (Intel processor Core i7‐2600 CPU@3.40 GHz).

##### Docking Studies

The molecular structures of compounds **(** ***R*** **)‐1d** and **(** ***S*** **)‐1d** were built by MOE2009.10 (builder module), parameterized by MMFF94x force field, and their docking poses within USP‐7 were calculated by Autodock 4.2.^[^ ^31^ ^]^ After the removal of water molecules and ligand atoms from the crystal structure of USP‐7N terminal domain (PDB ID: 5N9T),^[^ ^32^ ^]^ polar hydrogen and Gasteiger–Huckel charges were added. The ligand root was defined automatically. A 60 × 60 × 60 Å grid (grid spacing 0.375 Å) was centered in the binding site of the cocrystalized ligand, and electrostatic and affinity maps for each atom type of the ligand were calculated. The docking search was performed over 100 conformers using the Genetic Algorithm Local Search protocol as implemented in Autodock (population size: 50; rate of gene mutation: 0.02; rate of crossover: 0.8). The docking poses were clustered (rmsd: 2.0 Å) and the best conformation of the low‐energy highest populated cluster was selected as the binding conformation. Model analysis was carried out using the CCP4 program suite.^[^ ^39^ ^]^

##### Molecular Dynamics Studies

The coordinates of **(** ***S*** **)‐1d** docking pose were parameterized by CHARMM‐GUI.^[^ ^40^ ^]^ The molecular structure of the USP‐7 enzyme was processed and parameterized by the VMD package version 1.9.3 (Automatic PSF building module).^[^ ^41^ ^]^ The protein–ligand complex was assembled, and a solvation box including the whole complex (max box padding: *X* = 7; *y* = 0 7, *z* = 7) was defined. The molecular dynamics simulation was carried out by the software NAMD 2.14, developed by the Theoretical and Computational Biophysics Group in the Beckman Institute for Advanced Science and Technology at the University of Illinois at Urbana‐Champaign.^[^ ^42^ ^]^ The following parameters were adopted: run = 5,010,000; time step = 2; temperature = 310; restartfreq = 50. VMD “RMSD trajectory tool” and “Hydrogen bonds” were used to analyze the trajectory file.

## Conflict of Interest

The authors declare no conflict of interest.

## Author Contributions

**Matteo Lusardi**: investigation (lead); writing—review and editing (Equal). **Elva Morretta**: data curation (equal); investigation (equal); methodology (equal). **Andrea Spallarossa**: investigation (equal); methodology (equal); writing—review and editing (equal). **Maria Chiara Monti**: methodology (equal); supervision (equal); writing—review and editing (equal). **Camillo Rosano**: supervision (supporting); **Erika Iervasi**: data curation (equal); methodology (equal). **Marco Ponassi**: investigation (equal); methodology (equal); supervision (equal). **Matteo Mori**: investigation (equal); methodology (equal); writing—review and editing (equal). **Fiorella Meneghetti**: methodology (equal); supervision (equal); writing—review and editing (equal). **Chiara Brullo**: conceptualization (lead); data curation (lead); investigation (lead); writing—original draft (lead).

## Supporting information

Supplementary Material

## Data Availability

The data that support the findings of this study are available from the corresponding author upon reasonable request.
