# Supplementary material for: Targeting Ubiquitin‐Specific Protease 7 with Novel 5‐Amino‐Pyrazole Inhibitors: Design, Synthesis, and Biological Evaluation
Source: ChemMedChem. 2025 Jun 24;20(16):e202500185. doi: 10.1002/cmdc.202500185 (PMC12368478; doi:10.1002/cmdc.202500185)

ChemMedChem

Supporting Information

Targeting USP-7 With Novel 5-Amino-Pyrazole Inhibitors: Design, Synthesis and Biological Evaluation

Matteo Lusardi,[a,b] Elva Morretta,[c] Andrea Spallarossa,[a] Maria Chiara Monti,[c] Camillo Rosano,[d] Erika Iervasi,[d] Marco Ponassi,[d] Matteo Mori,[e] Fiorella Meneghetti,[e] Chiara Brullo*[a]

[a]Department of Pharmacy, University of Genova, Viale Benedetto XV, 3, 16132 Genova, Italy

[b]Molecular Modeling and Drug Discovery Laboratory, Istituto Italiano di Tecnologia, Via Morego, 30, 16163, Genova, Italy.

[c]Department of Pharmacy, University of Naples "Federico II", Via Domenico Montesano, 49, 80131 Napoli, Italy.

[d]IRCCS Ospedale Policlinico San Martino, L.go. R. Benzi, 10 I-16132 Genova, Italy.

[e]Department of Pharmaceutical chemistry, University of Milan, Via Mangiagalli, 25, 20133 Milano, Italy.

* Corresponding author: chiara.brullo@unige.it

Table of contents

Table S1. Analysis of the intermolecular contacts on HS-1 of **1b**.
Table S2. Crystal data and structure refinement details for **1b**.
Table S3. Predicted pharmacokinetics and drug-like properties of compounds **1d** and **STIRUR-41.**
Table S4. Interatomic distances (cutoff distance = 4.00 Å) in the USP-7/1d(R) complex.
Table S5. Interatomic distances (cutoff distance = 4.00 Å) in the USP-7/1d(S) complex-
Figure S1. Dose response curve of compounds **1a-d** on USP-7 enzyme
Figure S2. ^{1}H (400 MHz, DMSO-d_6) spectrum of compound **1a.**
Figure S3. ^{13}C (101 MHz, DMSO-d_6) spectrum of compound **1a.**
Figure S4. ^{1}H (400 MHz, DMSO-d_6) spectrum of compound **1b.**
Figure S5. ^{13}C (101 MHz, DMSO-d_6) spectrum of compound **1b.**
Figure S6. ^{1}H (400 MHz, DMSO-d_6) spectrum of compound **1c.**
Figure S7. ^{13}C (101 MHz, DMSO-d_6) spectrum of compound **1c.**
Figure S8. ^{1}H (400 MHz, DMSO-d_6) spectrum of compound **1d.**
Figure S9. ^{13}C (101 MHz, DMSO-d_6) spectrum of compound **1d.**
Figure S10. ^{1}H (400 MHz, DMSO-d_6) spectrum of compound **2a.**
Figure S11. ^{13}C (101 MHz, DMSO-d_6) spectrum of compound **2a.**
Figure S12. ^{1}H (400 MHz, DMSO-d_6) spectrum of compound **2b.**
Figure S13. ^{13}C (101 MHz, DMSO-d_6) spectrum of compound **2b.**
Figure S14. ^{1}H (400 MHz, DMSO-d_6) spectrum of compound **2c.**
Figure S15. ^{13}C (101 MHz, DMSO-d_6) spectrum of compound **2c.**
Figure S16. ^{1}H (400 MHz, DMSO-d_6) spectrum of compound **2d.**
Figure S17. ^{13}C (101 MHz, DMSO-d_6) spectrum of compound **2d.**

Table S1. Analysis of the intermolecular contacts on HS-1 of **1b**. Contact values were computed with *CrystalExplorer*. Surface contributions and enrichment ratios were calculated according to Jelsch.

**Atoms**	**H**	**C**	**N**	**O**	**F**
Surface (%)	70.3	11.1	4.8	8.6	5.5
Contacts (%)					
H	47.0				
C	13.4	3.4			
N	8.0	0.5	0.4		
O	15.7	1.2	0.2	0	
F	9.5	0.2	0	0	0.6
Enrichments					
H	0.95				
C	0.86	**2.76**			
N	**1.19**	0.50	-		
O	**1.30**	0.63	-	-	
F	**1.23**	0.16	-	-	-

Table S2. Crystal data and structure refinement details for **1b**.

Identification code	**1b**
Empirical formula (sum)	$C_{32}H_{38}F_2N_8O_8$
Formula weight (sum)	700.70
Temperature (K)	293(2)
Wavelength (Å)	0.71073
Crystal system	Orthorhombic
Space group	$Pca2_1$
Unit cell dimensions (Å/°)	a = 13.5011(6)<br>b = 30.4561(14)<br>c = 8.6895(4)
Volume (Å^3)	3573.0(3)
Z	4
Density calcd. (Mg/m^3)	1.303
Abs. coefficient (mm^{-1})	0.102
F(000)	1472
Crystal size (mm^3)	0.7 x 0.2 x 0.02
ϑ range data collection (°)	1.650 to 26.372
Index ranges	$-13 \leq h \leq 16$, $-38 \leq k \leq 37$, $-6 \leq l \leq 10$
Reflections collected	21414
Independent reflections	6268 [R_{int} = 0.0411]
Completeness to ϑ_{max} (%)	99.9
Refinement method	Full-matrix least-squares on F^2
Data/restraints/parameters	6268/1/451
Goodness-of-fit on F^2	1.021
Final R indices [$I>2\sigma(I)$]	R1 = 0.0462, wR2 = 0.1114
R indices (all data)	R1 = 0.0800, wR2 = 0.1306
Flack parameter	-0.2(7)
Largest diff. peak/hole (e·Å^{-3})	0.167 and -0.159
CCDC deposition number	2421400

Table S3. Predicted pharmacokinetics and drug-like properties of compounds **1d** and **STIRUR-41**

	**STIRUR-41**	**1d**
**Physicochemical Prop.**		
MW (g/mol)	350.34	400.35
Fraction Csp^3	0.31	0.35
Rotatable bonds	9	10
H-bond acceptors	6	8
H-bond donors	3	2
TPSA[a] ($Å^2$)	105.48	108.47
**Lipophilicity**		
LogP[b]	1.68	3.18
**Water solubility**		
Solubility (mg/mL)[c]	5.53×10^{-1}	4.43×10^{-2}
Solubility class	soluble	soluble
**Pharmacokinetics**		
GI absorption	hight	hight
BBB permeant	no	no
Pgp substrate	yes	no
CYP1A2 inhibitor	no	yes
CYP2C19 inhibitor	no	yes
CYP2C9 inhibitor	no	yes
CYP2D6 inhibitor	no	no
CYP3A4 inhibitor	no	no
**Druglikeness**		
Lipinski violations	0	0
**Medicinal chemistry**		
PAINS alerts	0	0
Brenk alerts	0	1

[a] Topological Polar Surface Area. [b] Predicted according to XLOGP3 program. [c] Values predicted by ESOL method.

Table S4. Interatomic distances (cutoff distance = 4.00 Å) in the USP-7/1d(R) complex. *** = H bond

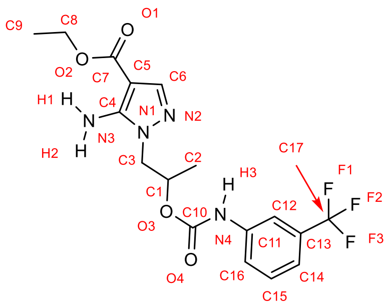

LIGAND ATOM	RESIDUE/ATOM	DISTANCE (Å)
C1	295(ASP)/OD1	3.29
	295(ASP)/CG	3.92
	297(GLN)/OE1	3.92
C2	405(GLN)/CB	3.25
	405(GLN)/CG	3.73
	406(LEU)/O	3.80
	297(GLN)/OE1	3.40
	297(GLN)/NE2	3.93
C3	296(VAL)/CG1	3.52
	295(ASP)/OD1	3.90
N1	296(VAL)/CG1	3.71
	296(VAL)/N	3.51
	295(ASP)/OD1	3.46
C4	295(ASP)/OD1	2.94
C5	295(ASP)/OD1	3.73
C6	224(TYR)/CD2	3.26
	224(TYR)/CE2	3.36
	296(VAL)/N	3.35
	296(VAL)/CB	3.90
N2	296(VAL)/CG1	3.19
	295(ASP)/C	3.99
	296(VAL)/N	2.96
	296(VAL)/CA	3.57
	296(VAL)/CB	3.11
N3	409(PHE)/CB	3.64
	295(ASP)/OD1	2.68
	409(PHE)/CD2	3.68
	295(ASP)/CG	3.90
H1	409(PHE)/CB	3.75
	295(ASP)/OD1	1.95
	409(PHE)/CD2	3.89
	295(ASP)/CG	3.13
	295(ASP)/OD2	3.90
H2	409(PHE)/CB	3.03
	409(PHE)/CG	3.63
	295(ASP)/OD1	3.57

	409(PHE)/CD2	3.62
C7	294(HIS)/O	3.93
	461(HIS)/NE2	3.28
O1	461(HIS)/CE1	3.23
	461(HIS)/CD2	3.53
	461(HIS)/NE2	2.66 ***
O2	224(TYR)/CE2	3.82
	291(PHE)/O	3.60
	294(HIS)/C	3.71
	294(HIS)/O	2.74
	461(HIS)/NE2	3.14
C8	461(HIS)/CE1	3.74
	291(PHE)/O	3.80
	294(HIS)/C	3.73
	294(HIS)/O	3.05
	461(HIS)/NE2	3.24
	292(MET)/C	3.83
	292(MET)/O	3.18
C9	294(HIS)/C	3.51
	294(HIS)/O	3.28
	295(ASP)/N	3.72
	295(ASP)/CA	3.91
	292(MET)/O	3.88
	295(ASP)/OD1	3.19
	295(ASP)/CG	3.77
O3	409(PHE)/CB	3.94
	406(LEU)/O	3.77
	295(ASP)/OD1	3.77
C10	409(PHE)/N	3.57
	409(PHE)/CB	3.45
	406(LEU)/O	3.88
O4	407(MET)/C	3.35
	408(ARG)/N	2.99
	408(ARG)/CA	3.96
	408(ARG)/C	3.92
	409(PHE)/N	3.03
	409(PHE)/CA	3.85
	409(PHE)/CB	3.77
	406(LEU)/C	3.96
	406(LEU)/O	3.17
	407(MET)/CA	3.12
N4	409(PHE)/CB	3.39
	295(ASP)/OD1	3.09
	295(ASP)/CG	3.83
	295(ASP)/OD2	4.00
	409(PHE)/O	3.85
H3	409(PHE)/CB	3.64

	295(ASP)/OD1	2.15 ***
	295(ASP)/CG	3.04
	295(ASP)/OD2	3.41
C11	409(PHE)/CB	3.73
	295(ASP)/OD1	3.82
	409(PHE)/O	3.10
C12	295(ASP)/OD1	3.85
	295(ASP)/OD2	3.80
	409(PHE)/O	3.21
C13	409(PHE)/O	3.28
C14	409(PHE)/O	3.27
C15	409(PHE)/O	3.17
C16	409(PHE)/C	4.00
	409(PHE)/O	3.08
C17	418(ASN)/ND2	3.43
	418(ASN)/CG	3.84
	418(ASN)/OD1	3.74
F1	418(ASN)/ND2	3.01
	418(ASN)/CG	3.14
	418(ASN)/OD1	2.70
F3	418(ASN)/ND2	2.65
	418(ASN)/CG	3.45
	418(ASN)/OD1	3.78

Table S5. Interatomic distances (cutoff distance = 4.00 Å) in the USP-7/1d(S) complex. *** = H bond

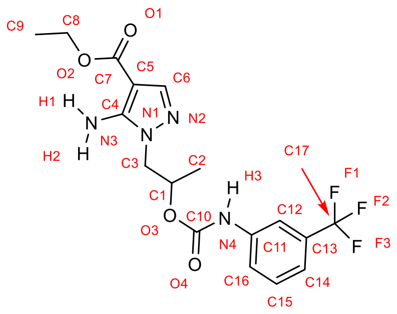

LIGAND ATOM	RESIDUE/ATOM	DISTANCE (Å)
C1	295(ASP)/OD1	3.88
C2	405(GLN)/CB	3.48
	405(GLN)/CG	3.96
	406(LEU)/O	3.71
	297(GLN)/OE1	3.60
C3	296(VAL)/CG1	3.51
	295(ASP)/CA	3.84
	296(VAL)/N	3.43
	295(ASP)/OD1	3.00
	295(ASP)/CB	3.46
	295(ASP)/CG	3.57
	297(GLN)/OE1	3.46
N1	296(VAL)/CG1	3.91
	295(ASP)/CA	3.84
	296(VAL)/N	3.38
	295(ASP)/OD1	3.25
	295(ASP)/CB	3.99
C4	295(ASP)/OD1	3.14
C6	224(TYR)/CD2	3.38
	224(TYR)/CE2	3.72
N2	296(VAL)/CG1	3.44
	296(VAL)/N	3.20
	296(VAL)/CA	3.80
	296(VAL)/CB	3.33
N3	409(PHE)/CB	3.67
	409(PHE)/CG	3.92
	295(ASP)/OD1	2.66
	409(PHE)/CD2	3.44
	295(ASP)/CG	3.89
H1	409(PHE)/CB	3.99
	295(ASP)/OD1	1.74
	409(PHE)/CD2	3.56
	295(ASP)/CB	3.93
	295(ASP)/CG	2.98
	295(ASP)/OD2	3.80
H2	409(PHE)/CB	2.94

	409(PHE)/CG	3.50
	295(ASP)/OD1	3.24
	409(PHE)/CD2	3.38
C7	461(HIS)/NE2	3.43
O1	461(HIS)/CE1	3.23
	461(HIS)/CD2	3.80
	461(HIS)/NE2	2.89
	409(PHE)/CE2	3.70
	409(PHE)/CD2	3.79
O2	465(TYR)/CE2	3.89
	465(TYR)/CZ	3.96
	465(TYR)/OH	3.10
	461(HIS)/CD2	3.64
	461(HIS)/NE2	3.52
C8	456(HIS)/CD2	3.90
	465(TYR)/CE2	3.86
	465(TYR)/CZ	3.89
	465(TYR)/OH	3.13
	456(HIS)/NE2	3.97
C9	465(TYR)/OH	3.25
	456(HIS)/CE1	3.89
	456(HIS)/NE2	3.59
	460(ASN)/C	3.80
	460(ASN)/O	3.80
	461(HIS)/N	3.98
O3	406(LEU)/O	3.94
	295(ASP)/OD1	3.95
C10	409(PHE)/N	3.73
	409(PHE)/CB	3.58
	406(LEU)/O	3.58
O4	407(MET)/C	3.53
	408(ARG)/N	2.82
	408(ARG)/CA	3.68
	408(ARG)/C	3.78
	409(PHE)/N	3.11
	409(PHE)/CB	3.80
	406(LEU)/C	3.69
	406(LEU)/O	2.66
	407(MET)/CA	3.44
N4	409(PHE)/CB	3.45
	295(ASP)/OD1	3.06
	295(ASP)/CG	3.88
H3	409(PHE)/CB	3.79
	295(ASP)/OD1	2.12 ***
	295(ASP)/CG	3.06
	295(ASP)/OD2	3.60
C11	409(PHE)/CB	3.55

	295(ASP)/OD1	3.69
	409(PHE)/O	3.38
C12	409(PHE)/CB	3.76
	295(ASP)/OD1	3.63
	295(ASP)/OD2	3.81
	409(PHE)/O	3.11
C13	409(PHE)/O	2.89
C14	409(PHE)/O	2.98
C15	409(PHE)/O	3.25
	407(MET)/CG	3.90
C16	409(PHE)/N	3.91
	407(MET)/CA	3.88
	409(PHE)/O	3.43
C17	418(ASN)/ND2	3.97
	409(PHE)/O	3.58
	418(ASN)/OD1	3.98
F2	418(ASN)/ND2	2.93
	409(PHE)/O	3.90
	418(ASN)/CG	3.44
	418(ASN)/OD1	3.65
F3	418(ASN)/ND2	3.74
	409(PHE)/O	3.55
	418(ASN)/CG	3.63
	418(ASN)/OD1	3.07

Figure S1. Dose response curve of compounds **1a-d** on USP-7 enzyme obtained through GraphPad Prism 7, were reported. Each data point is the average of three separate experiments.

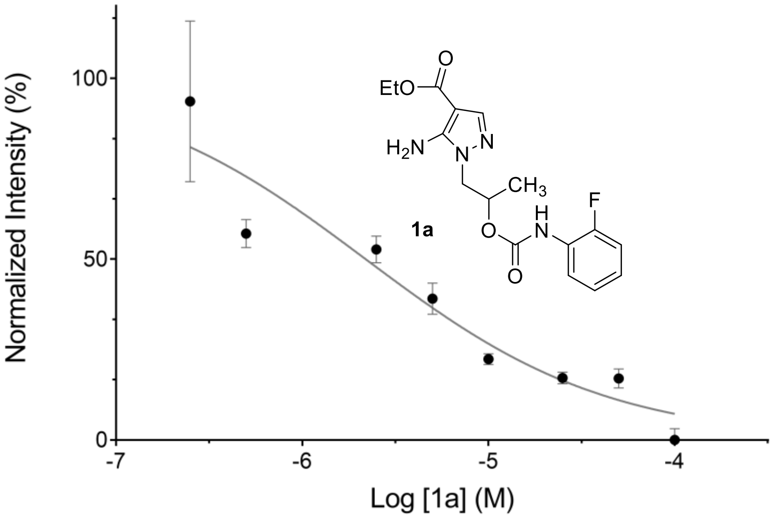

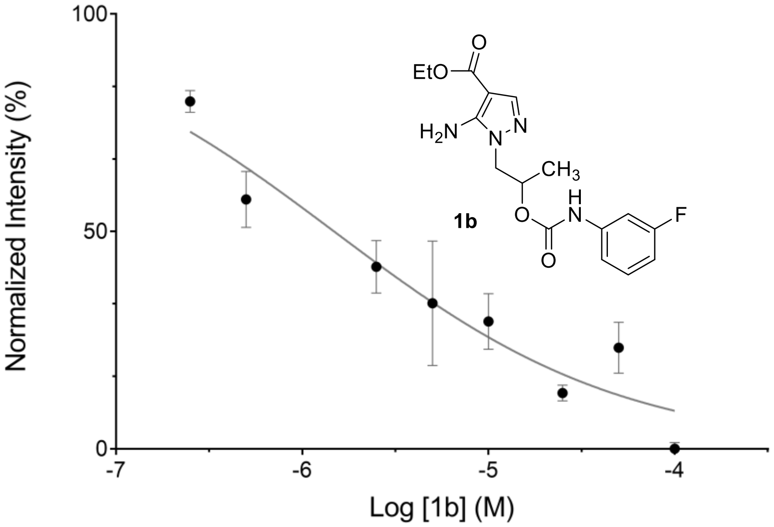

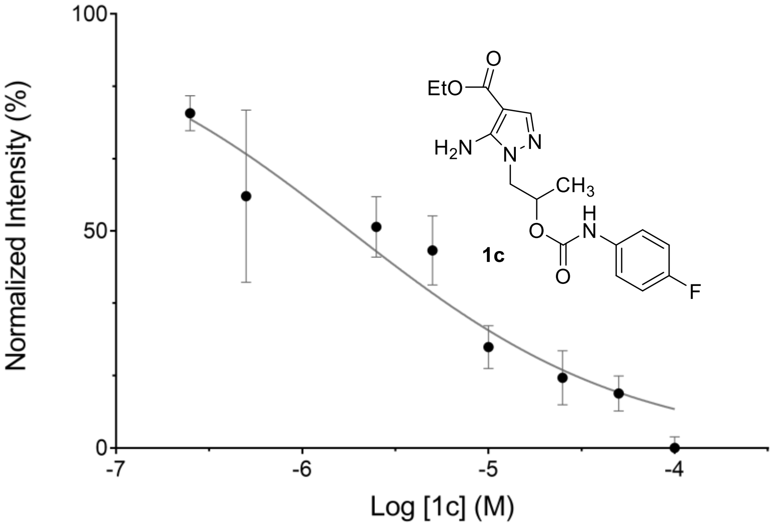

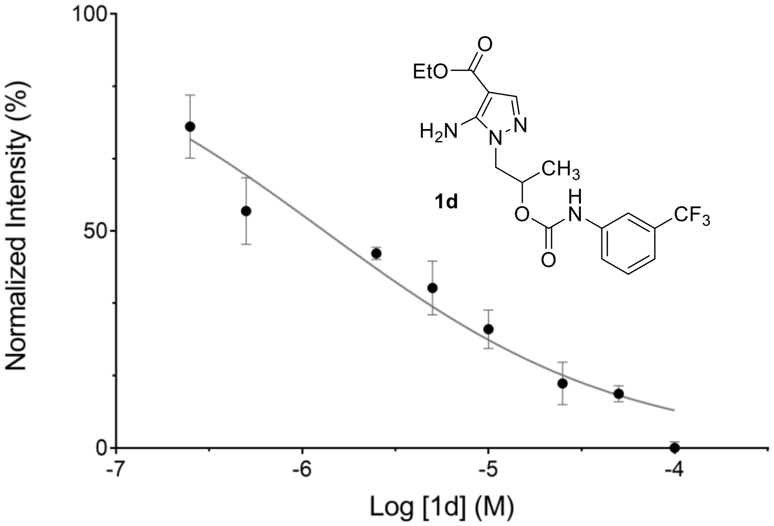

100
50
0
-7
-6
-5
-4
Normalized Intensity (%)
Log [1d] (M)
EtO
O
H2N
N
N
CH3
H
O
N
CF3
O
1d

Figure S2. ^{1}H (400 MHz, DMSO-d_6) spectrum of compound **1a.**

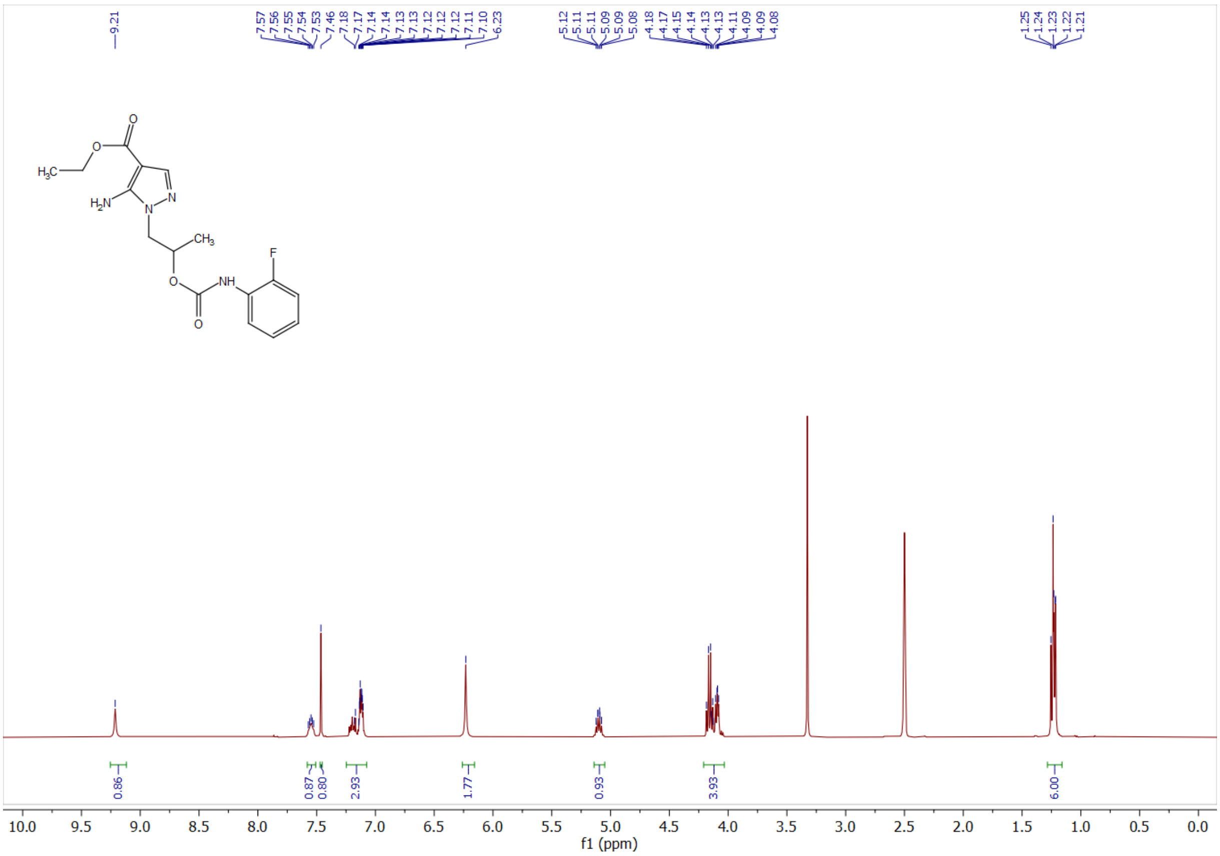

Figure S3. ^{13}C (101 MHz, DMSO-d$_6$) spectrum of compound **1a.**

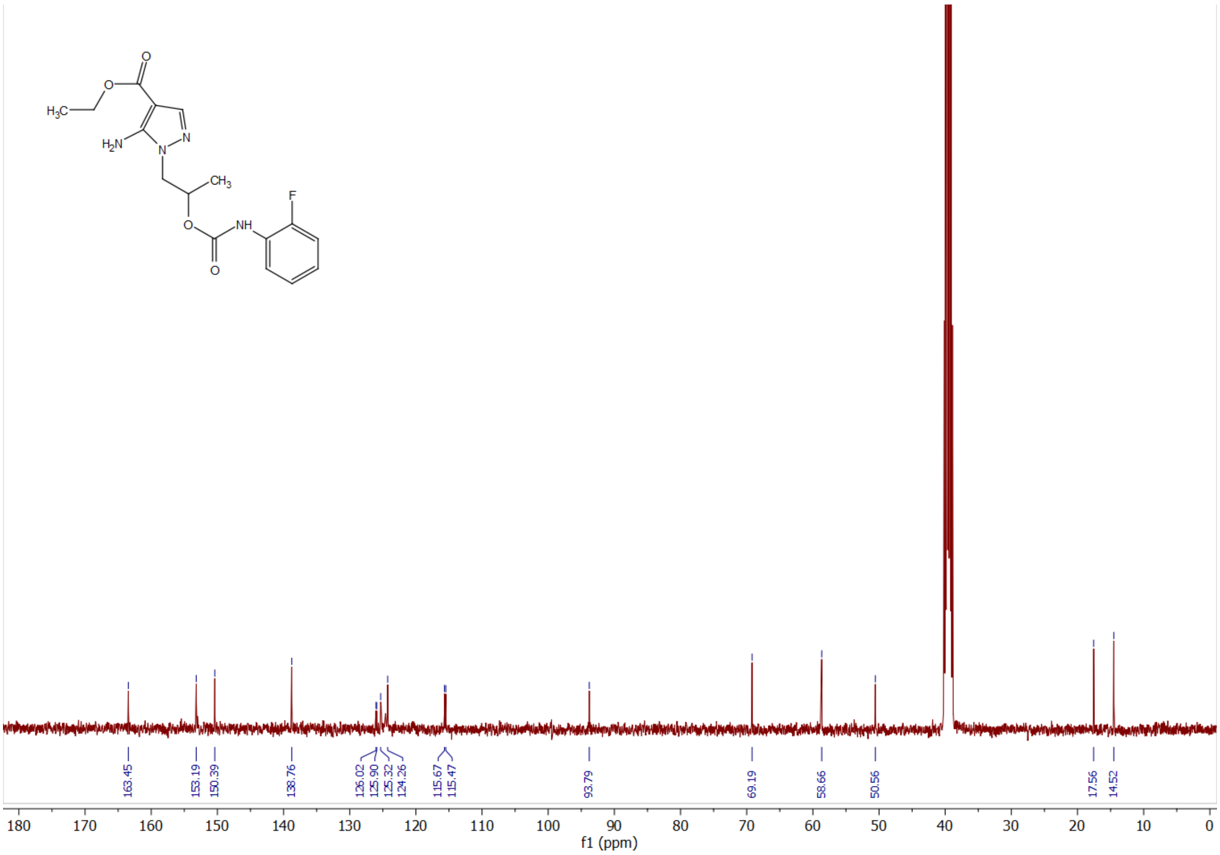

Figure S4. ^{1}H (400 MHz, DMSO-d$_6$) spectrum of compound **1b**

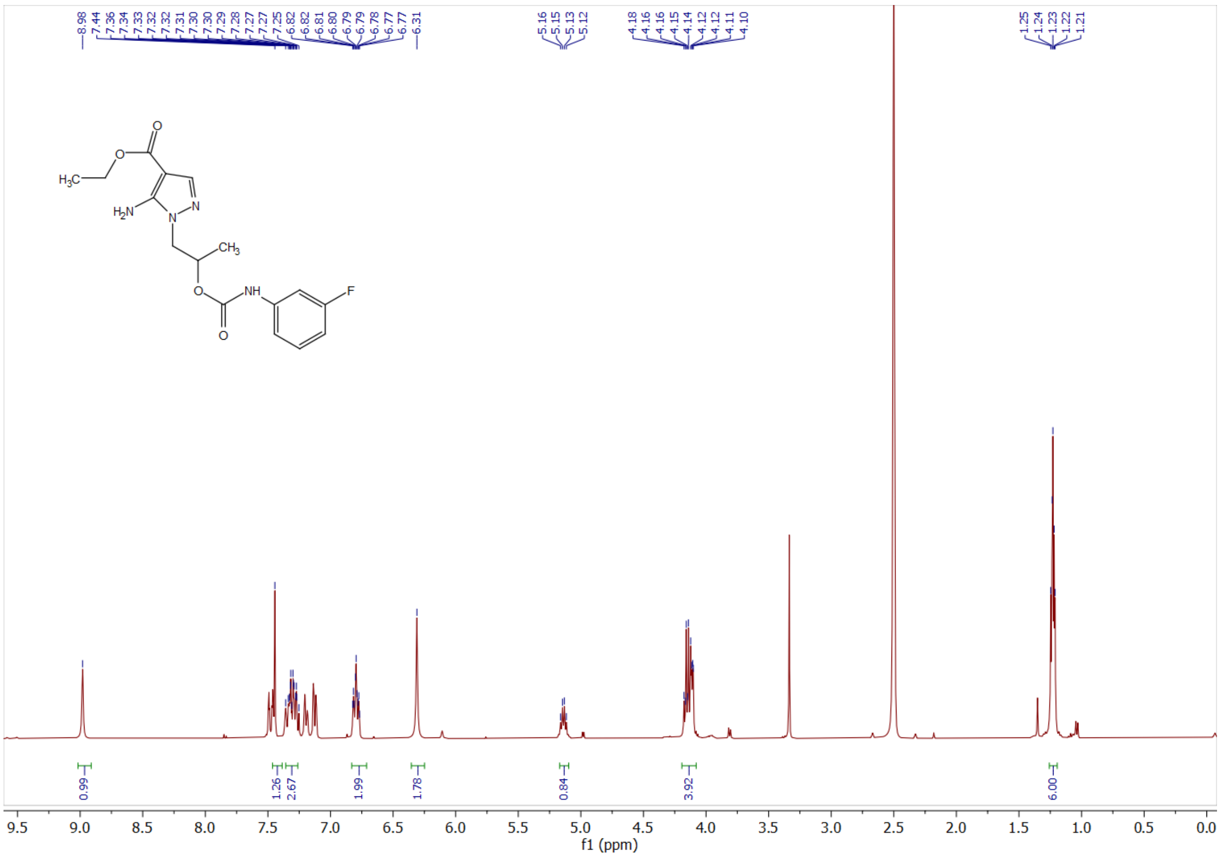

Figure S5. ^{13}C (101 MHz, DMSO-d_6) spectrum of compound **1b.**

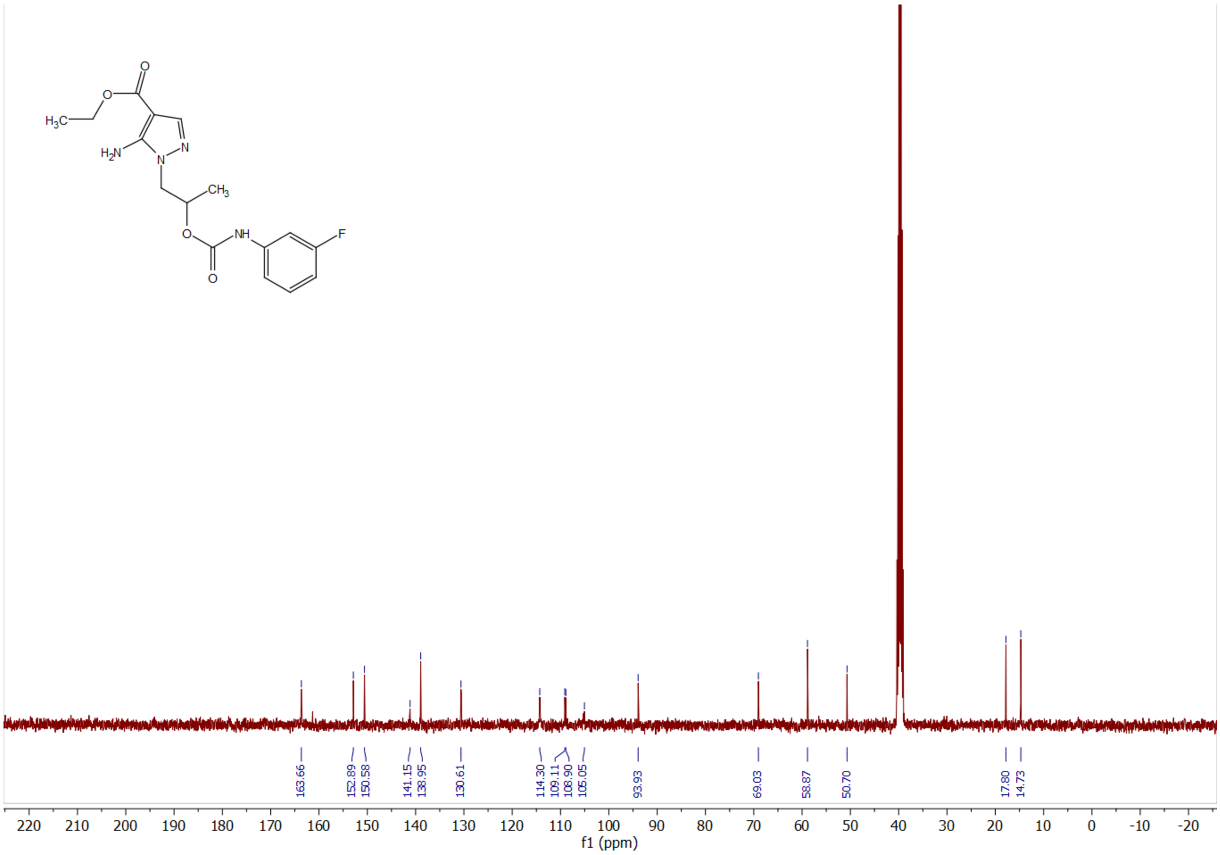

Figure S6. ^{1}H (400 MHz, DMSO-d_6) spectrum of compound **1c.**

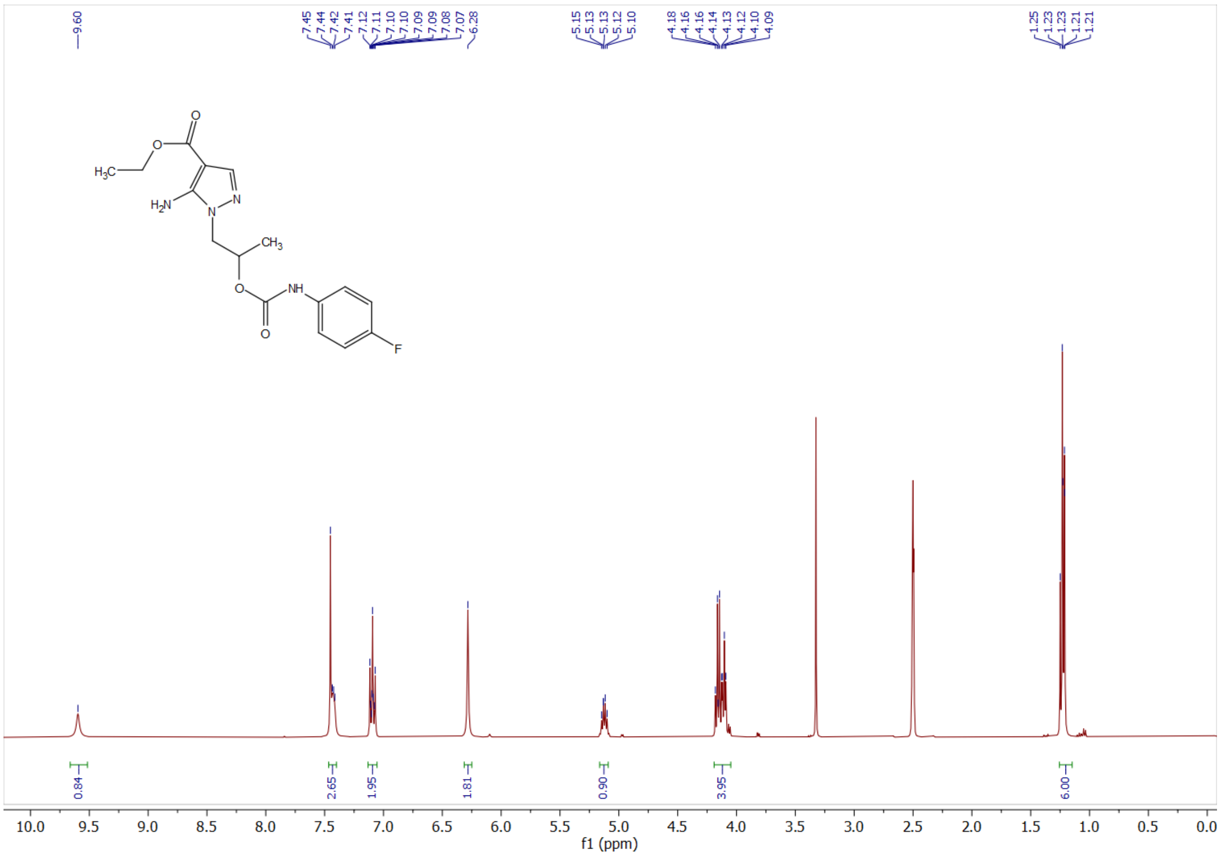

Figure S7. ^{13}C (101 MHz, DMSO-d$_6$) spectrum of compound **1c.**

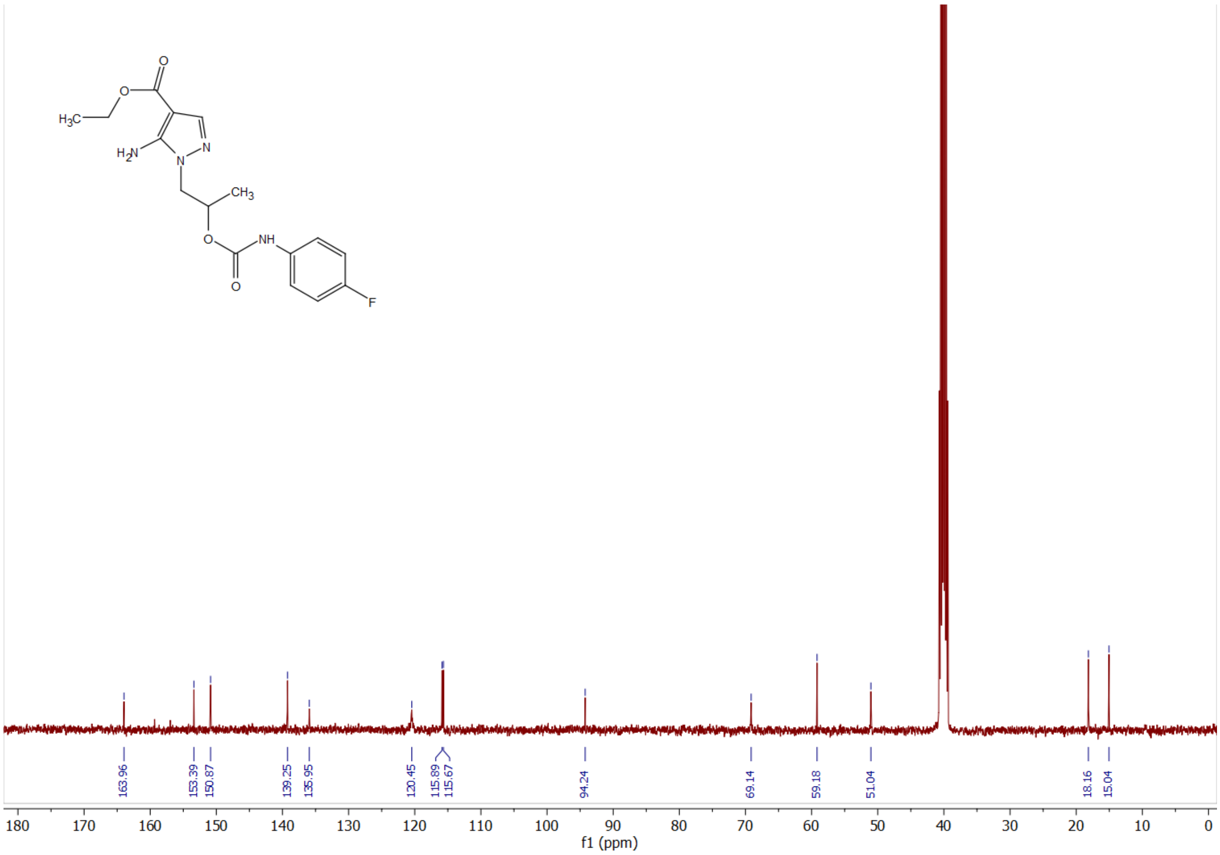

Figure S8. ^{1}H (400 MHz, DMSO-d$_6$) spectrum of compound **1d.**

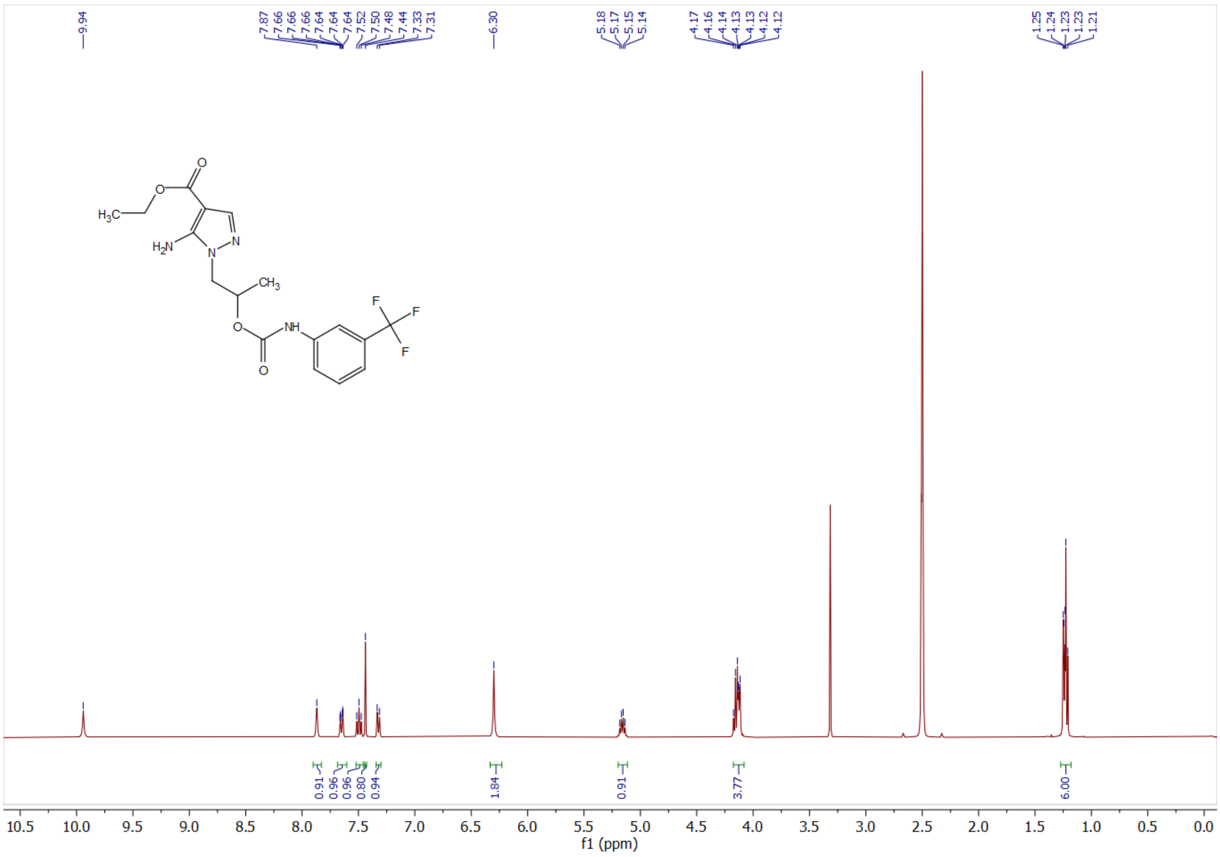

Figure S9. ^{13}C (101 MHz, DMSO-d$_6$) spectrum of compound **1d.**

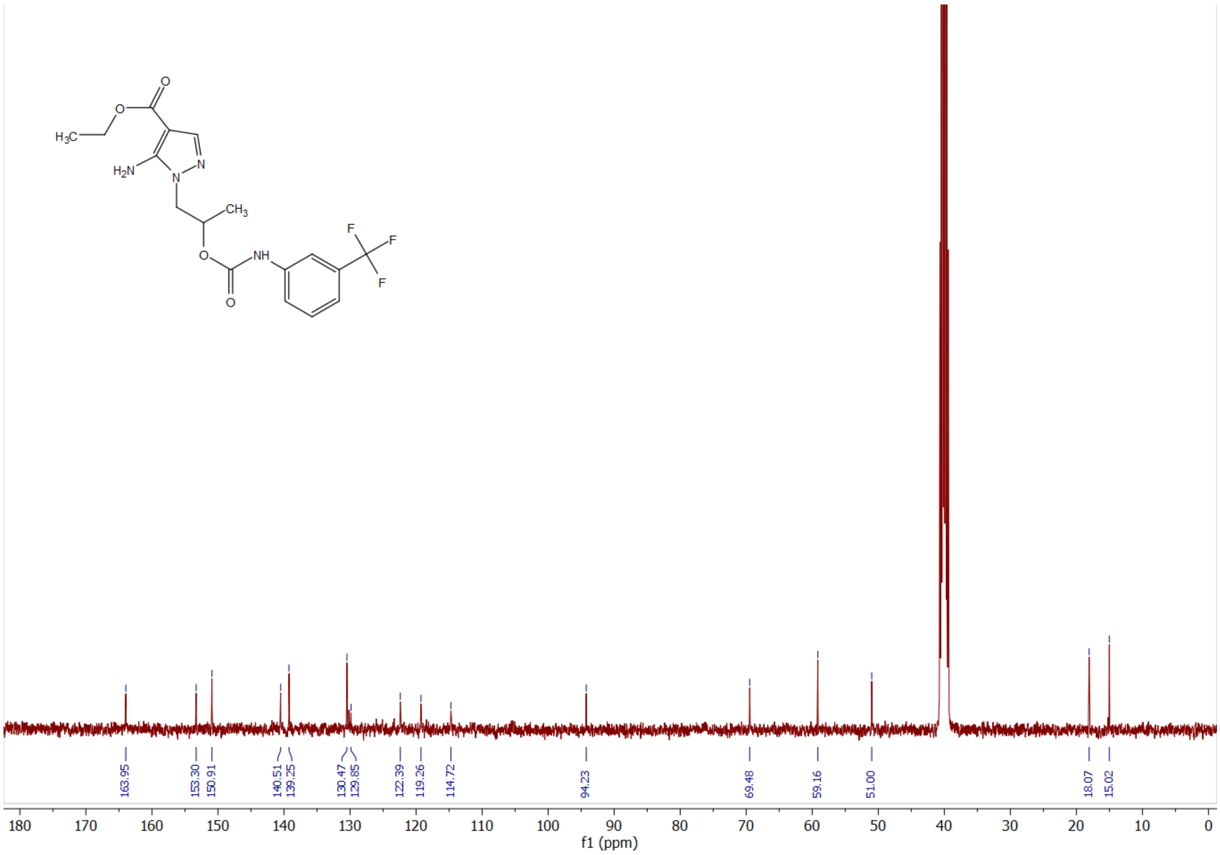

Figure S10. ^{1}H (400 MHz, DMSO-d$_6$) spectrum of compound **2a.**

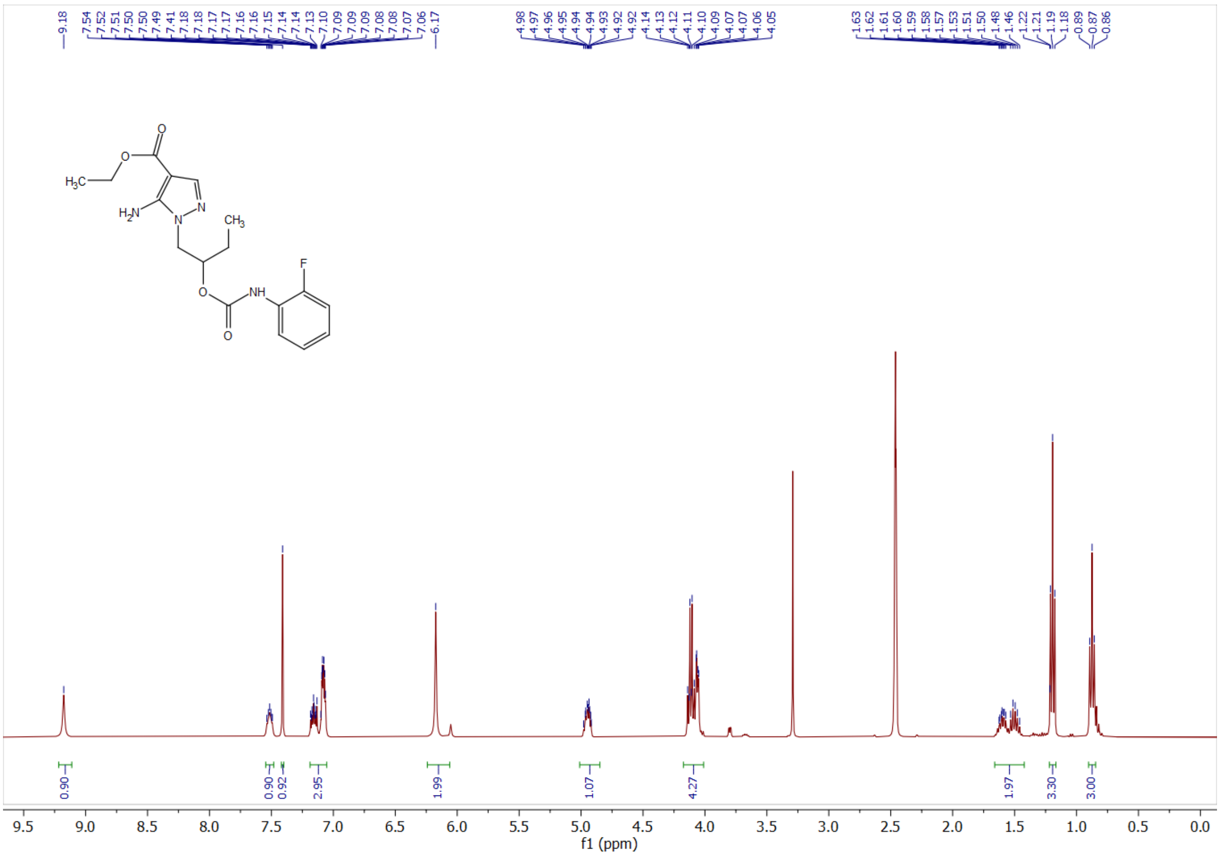

Figure S11. ^{13}C (101 MHz, DMSO-d_6) spectrum of compound **2a.**

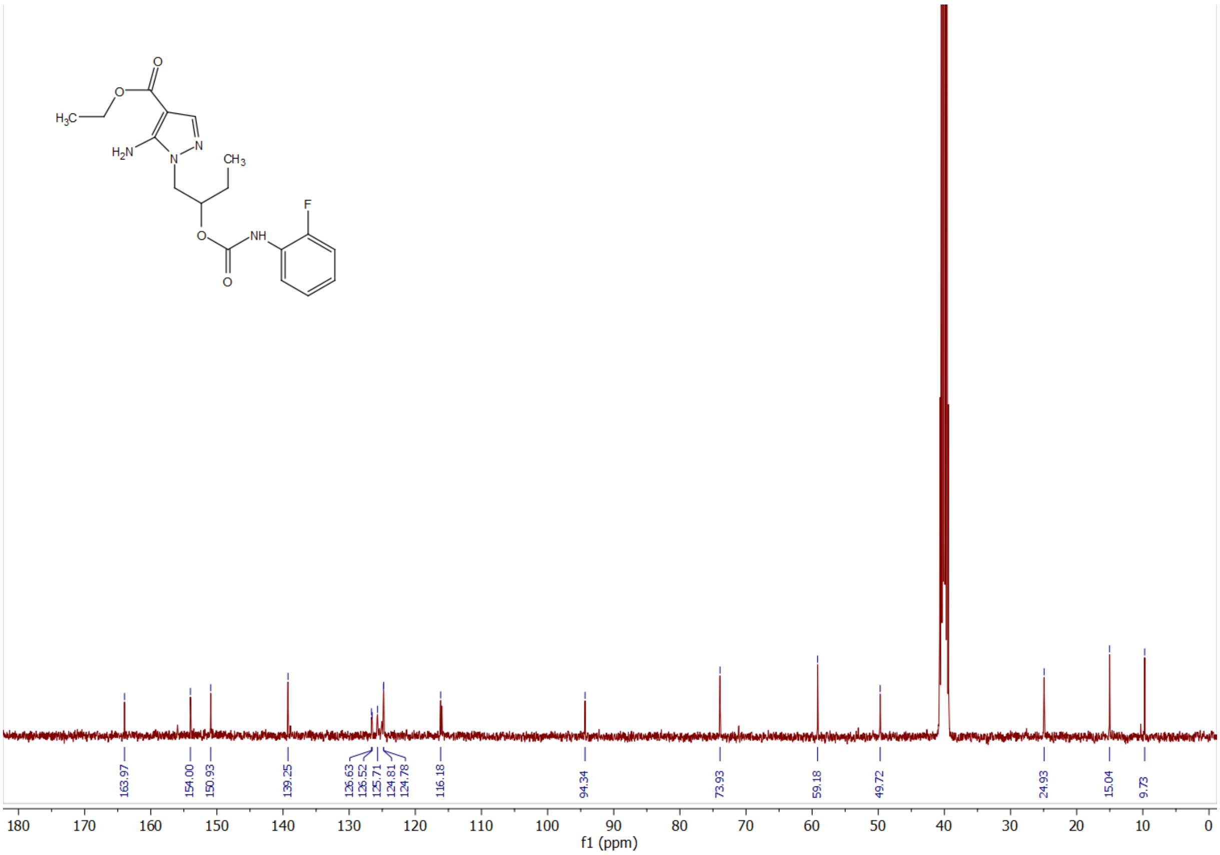

Figure S12. ^{1}H (400 MHz, DMSO-d_6) spectrum of compound **2b.**

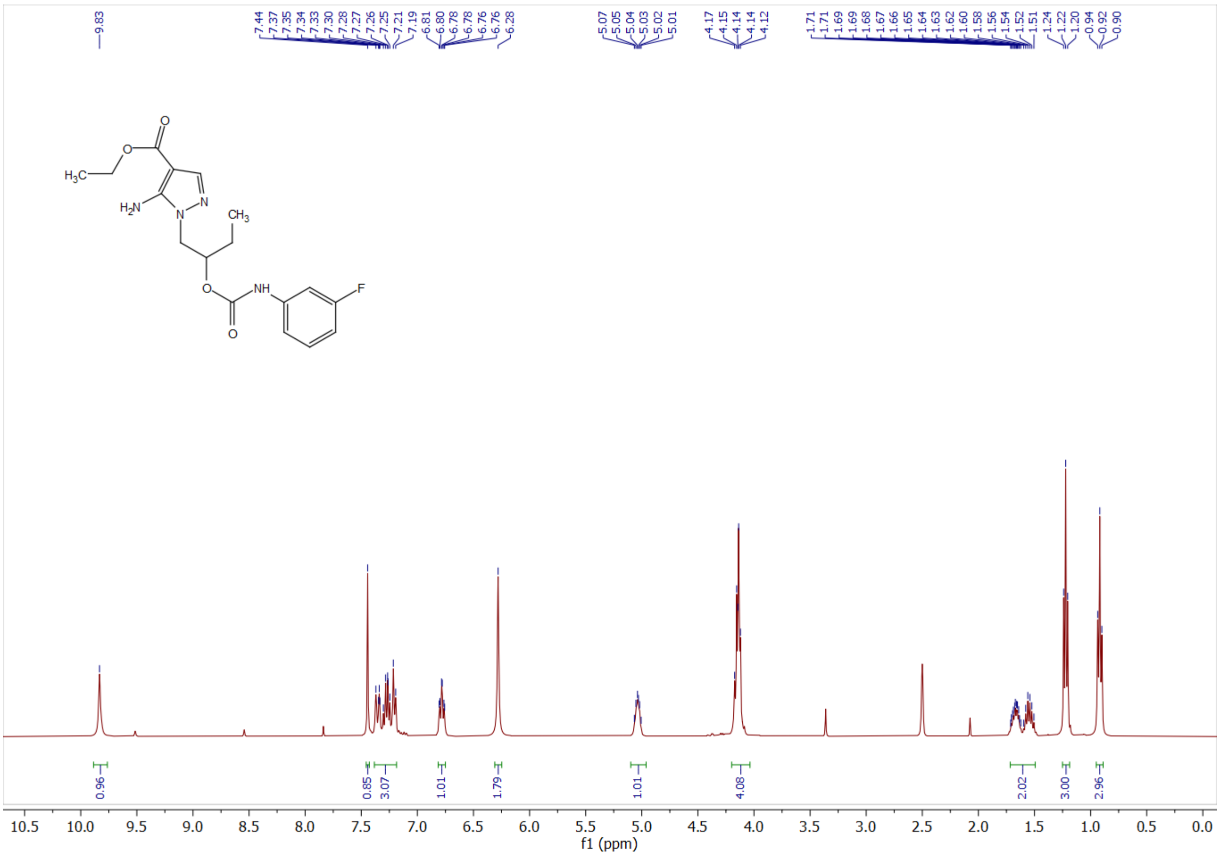

Figure S13. ^{13}C (101 MHz, DMSO-d_6) spectrum of compound **2b.**

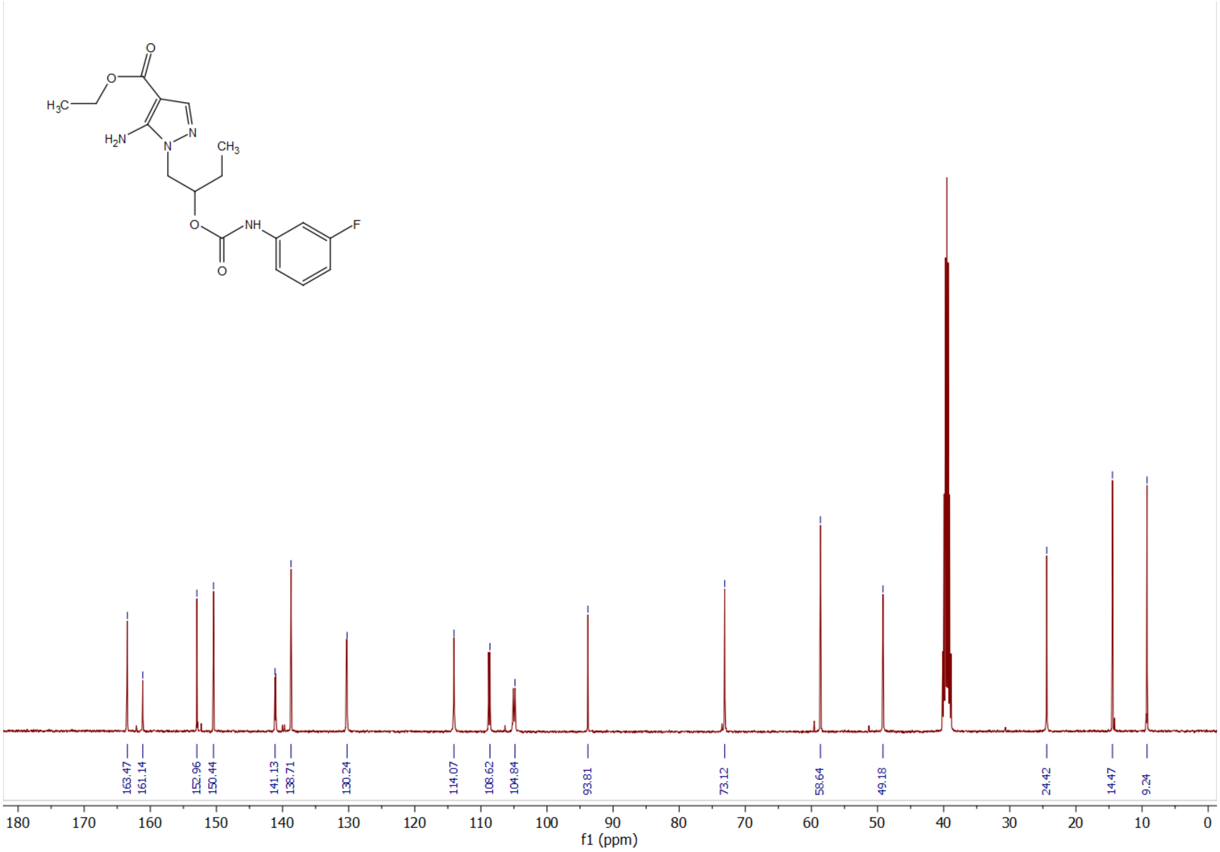

Figure S14. ^{1}H (400 MHz, DMSO-d_6) spectrum of compound **2c.**

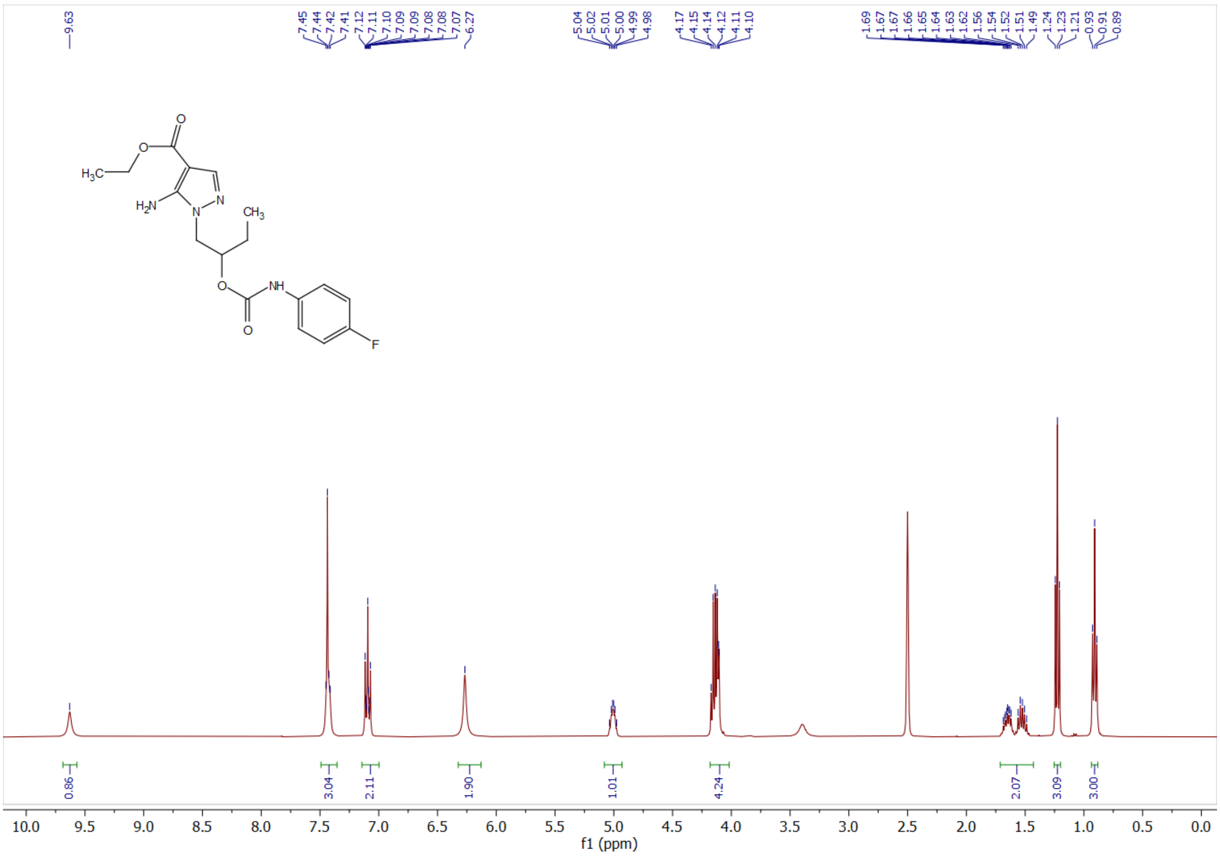

Figure S15. ^{13}C (101 MHz, DMSO-d_6) spectrum of compound **2c.**

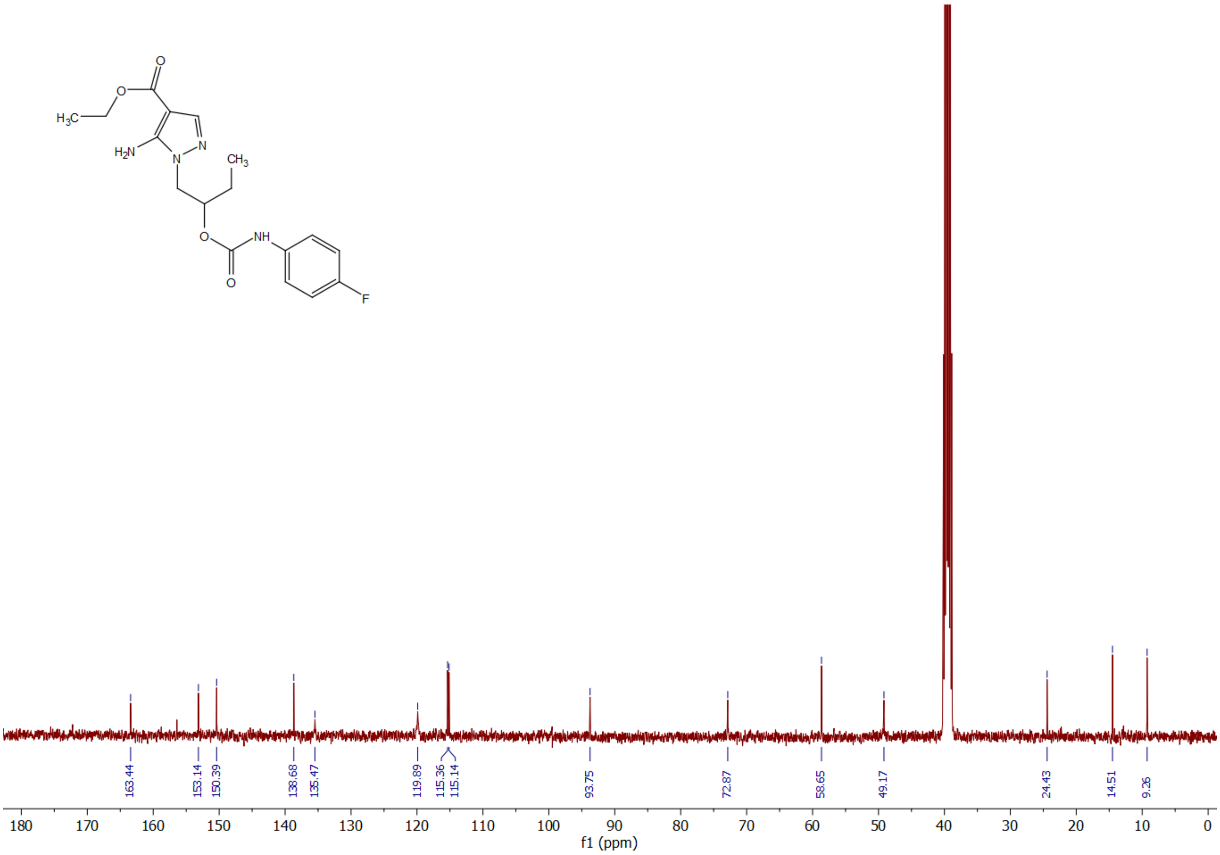

Figure S16. ^{1}H (400 MHz, DMSO-d_6) spectrum of compound **2d.**

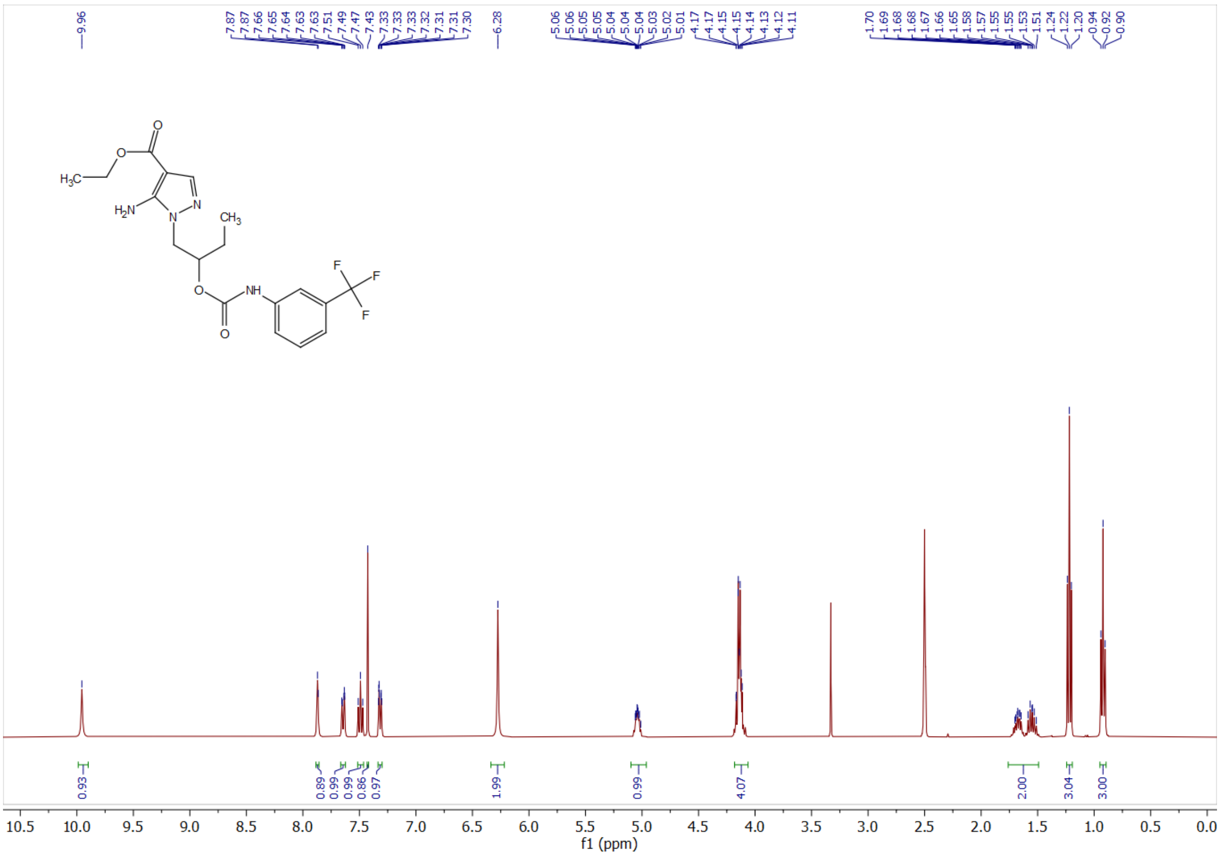

Figure S17. ^{13}C (101 MHz, DMSO-d_6) spectrum of compound **2d**

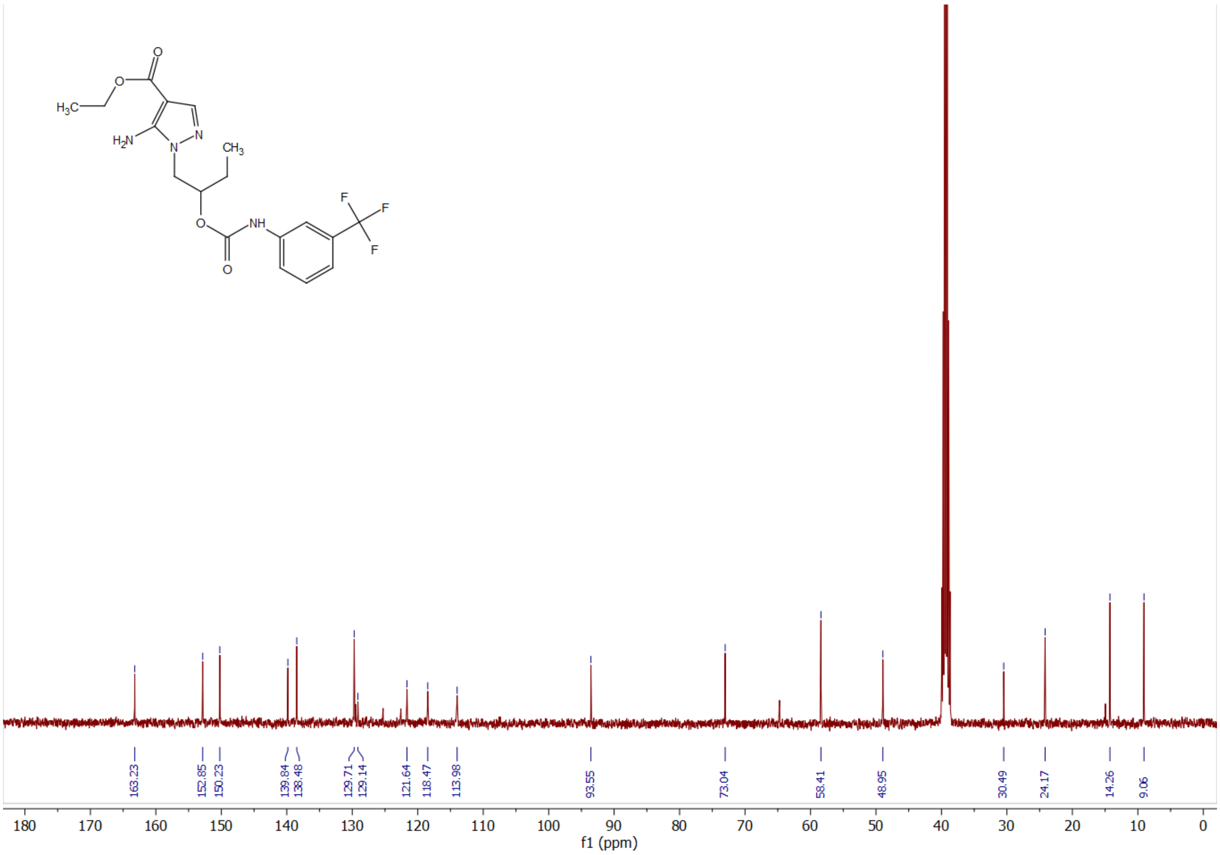

Supplement: Supplementary file 1 — Supplementary Material [file CMDC-20-e202500185-s001.pdf]
